# Two-Dimensional Quantum Hall Effect and Zero Energy State in Few-Layer ZrTe_5_

**DOI:** 10.1021/acs.nanolett.1c00958

**Published:** 2021-07-12

**Authors:** Fangdong Tang, Peipei Wang, Mingquan He, Masahiko Isobe, Genda Gu, Qiang Li, Liyuan Zhang, Jurgen H. Smet

**Affiliations:** † Max Planck Institute for Solid State Research, Stuttgart 70569, Germany; ‡ Department of Physics and Shenzhen Institute for Quantum Science and Engineering, Southern University of Science and Technology, Shenzhen 518055, China; § Low Temperature Physics Laboratory, College of Physics and Center of Quantum Materials and Devices, Chongqing University, Chongqing 401331, China; ∥ Condensed Matter Physics and Materials Science Division, Brookhaven National Laboratory, Upton, New York 11973-5000, United States; ⊥ Department of Physics and Astronomy, Stony Brook University, Stony Brook, New York 11794-3800, United States

**Keywords:** ZrTe_5_ thin film, 2D quantum Hall effect, zero energy state, topological insulator

## Abstract

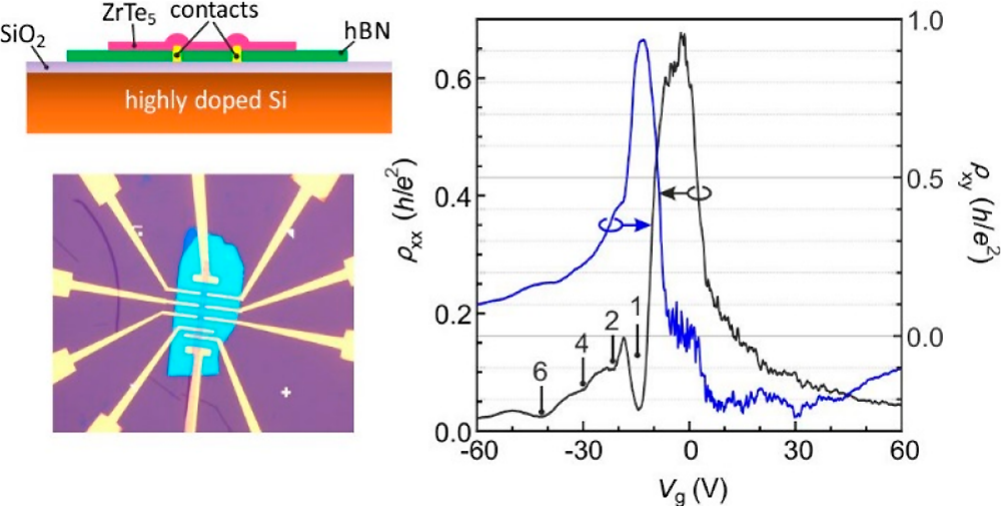

Topological matter plays a central role in today’s condensed matter research. Zirconium pentatelluride (ZrTe_5_) has attracted attention as a Dirac semimetal at the boundary of weak and strong topological insulators (TI). Few-layer ZrTe_5_ is anticipated to exhibit the quantum spin Hall effect due to topological states inside the band gap, but sample degradation inflicted by ambient conditions and processing has so far hampered the fabrication of high quality devices. The quantum Hall effect (QHE), serving as the litmus test for 2D systems to be considered of high quality, has not been observed so far. Only a 3D variant on bulk was reported. Here, we succeeded in preserving the intrinsic properties of thin films lifting the carrier mobility to ∼3500 cm^2^ V^–1^ s^–1^, sufficient to observe the integer QHE and a bulk band gap related zero-energy state. The magneto-transport results offer evidence for the gapless topological states within this gap.

ZrTe_5_ has recently emerged as an intriguing topological material hosting interesting quantum phases and correlation effects.^1−5^ The experimental observations of the chiral magnetic effect,^6^ the anomalous Hall effect,^7^ pressure driven superconductivity^8^ and excellent thermoelectric properties^9^ have attracted broad interest. It is an orthorhombic layered material belonging to the *Cmcm* space group. In the monolayer form, this should result in a quantum spin Hall insulator (QSHI)^3,5^ with a bulk band gap up to 0.1 eV.^3,10−12^ Because this system is located at the boundary of various topological phases,^3^ even moderate disturbances in the lattice constant or carrier density and possibly also disorder details seem to cause significantly different experimental observations. The latter have instigated an intense debate on the nontrivial band topology of bulk ZrTe_5_ and whether it should be regarded as a Dirac semimetal,^6,13,14^ a strong,^12,15^ or a weak topological insulator.^10,11,16,17^ When moving from bulk to thin films, additional complexity is added because of the poor stability of ZrTe_5_ when exposed to ambient conditions and the increased sensitivity to the surface and disorder. Complex transport behavior as well as low mobility have been observed in thin films, whereas the intrinsic physics remained elusive.^18−21^ Here, significant advances in observing the intrinsic magneto-transport properties of thin ZrTe_5_ are reported by fabricating and measuring devices without any exposure to ambient air. Clean single band transport, incipient quantum Hall effect behavior, and evidence for a bulk energy gap in this promising quantum spin Hall insulator material are the outcome of these experiments.

Our studies were performed on high-quality single crystals of ZrTe_5_ synthesized using either the tellurium flux method or chemical vapor transport.^4,22^ Crystals are needlelike and devices were fabricated by transferring mechanically exfoliated thin films on top of buried contacts inside atomically flat hBN to achieve the best sample quality (Section 15, Supporting Information (SI)). The entire fabrication and measurement procedure avoided exposure to air, solvents, as well as elevated temperatures, all of which cause quality degradation (Section 6, SI). A sample schematic is illustrated in Figure 1a, and an optical image made up of a 6.5 ± 1.1 nm thick ZrTe_5_ layer is shown in panel Figure 1b. In the remainder, the magneto-transport properties of this sample are discussed. Results for samples with different thicknesses are covered in the SI.

**Figure 1:**
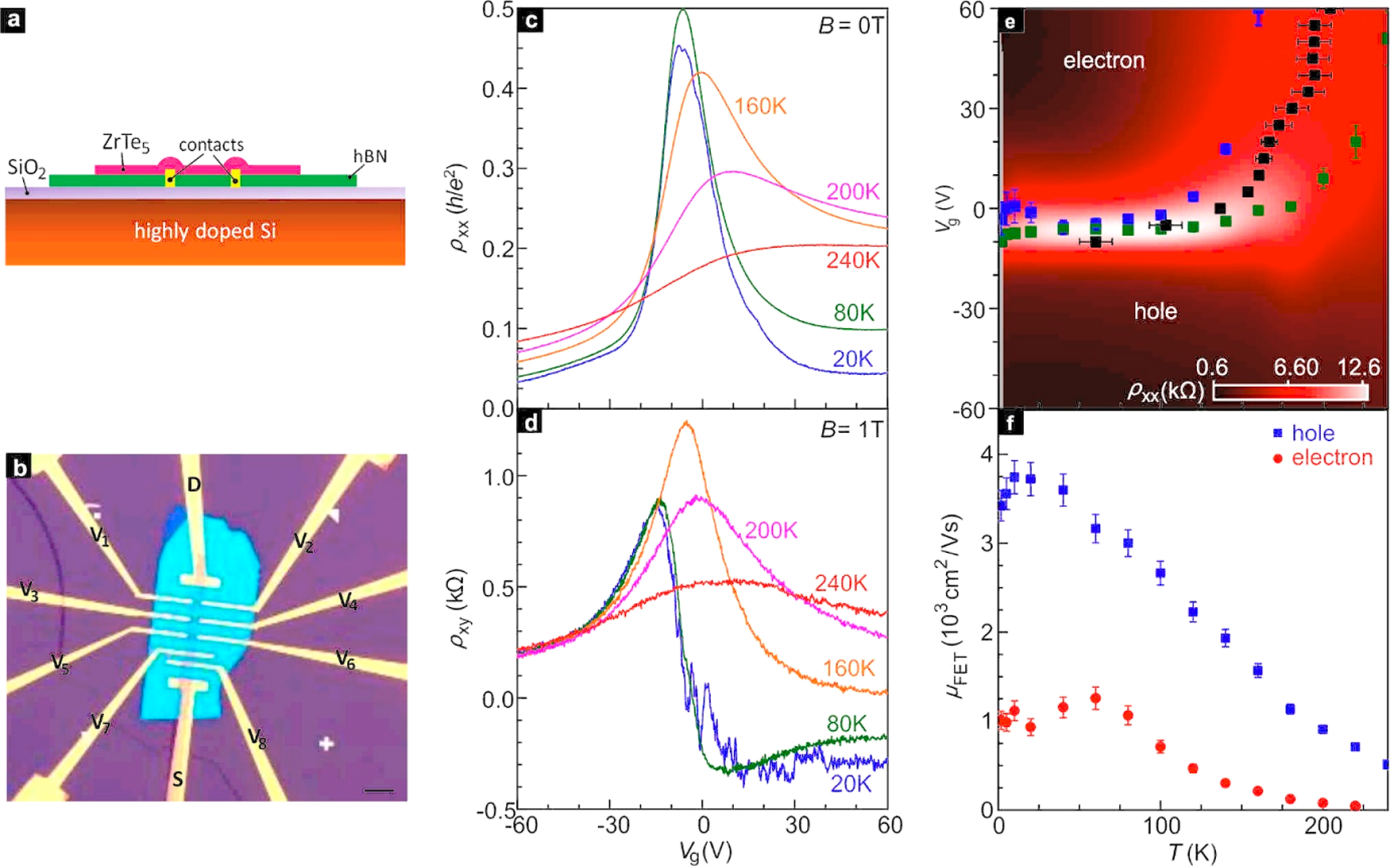
ZrTe_5_ device geometry and basic transport properties. (a) Schematic cross-sectional view of the device structure. (b) Optical image of the ZrTe_5_ device located on top of the hBN-substrate. The scale bar corresponds to 10 μm. The distance between adjacent voltage probes is about 6 μm. The width of the flake is about 3.3 μm and the distance between voltage probes on opposite sides is approximately equal to 2.1 μm. Current is sent from the source (S) to the drain (D). (c) Field effect traces of the longitudinal resistivity and (d) Hall resistivity at different temperatures. (e) Color map of the longitudinal resistivity across the plane spanned by gate voltage *V*_g_ and temperature *T.* The blue, black, and green square symbols mark the transition from hole to electron transport determined using three different criteria as described in the main text: the zero in the Hall resistivity (ρ_*xy*_(*V*_g_) = 0, blue), peak position in ρ_*xx*_ (*T*_p_, black), the sign reversal of the slope of ρ_*xx*_(*V*_g_) (dρ_*xx*_/d*V*_g_ = 0, green). (f) The temperature-dependent mobility of holes and electrons extracted for field effect traces.

Figure 1c,d illustrates field effect traces of the longitudinal resistivity, ρ_*xx*_, and Hall resistivity, ρ_*xy*_, between 20 and 240 K. A pronounced peak is observed in ρ_*xx*_ below 200 K. It is reminiscent of the charge neutrality peak in graphene^23,24^ and is attributed to the transition from majority hole transport in the valence band to majority electron transport in the conduction band as confirmed in ρ_*xy*_. However, above ∼220 K the Hall resistivity remains positive. Transport is apparently hole dominated at all back-gate voltages and the chemical potential resides deep inside the valence band. This behavior heralds the temperature-induced Lifshitz transition previously reported in the literature^4,13,16−18^ (Section 3 and 4, SI). Figure 1e displays a color map of ρ_*xx*_ in the (*V*_g*,*_*T*)-plane and confirms this interpretation. Two distinct regions can be identified separated by a fairly sharp boundary in the low-temperature regime that marks the transition from hole to electron transport. The transition is less well-defined at high temperatures where thermal excitation of charge carriers plays a role. Three criteria were adopted to determine this transition from hole to electron transport with less uncertainty. The Hall resistivity crossing through zero at a given *T* and *V*_g_ signals the charge inversion. Errors due to offset voltages were minimized by symmetrizing ρ_*xy*_. Exemplary raw data are shown in Figure 1d and in Figure S4b of the SI. The crossing points are included in Figure 1e as blue symbols. Also the maximum,  in the gate voltage-dependent ρ_*xx*_ data recorded at fixed temperature can be used as criterion (green symbols in Figure 1e, see also Figure S4c). Finally, the previously reported anomalous resistivity peak in the temperature dependence of ρ_*xx*_ is also a manifestation of charge carrier inversion that accompanies the temperature-induced Lifshitz transition of the Fermi surface^16^ (black symbols in Figure 1e and Figure S4a). At low temperature, all three criteria yield nearly the same transition point. However, data points drift apart for *T* > ∼200 K and *V*_g_ > ∼20 V. Below 100 K, the gate voltage where the transition occurs remains nearly fixed, yet it changes rapidly at higher temperature.^4,13^ For the sake of completeness, we note that the carrier density itself is not a good criterion as it changes rapidly with the temperature^4,13^ and gate voltage (Figure S2b, Supporting Information). The electron density can be tuned up to 10^12^ cm^–2^, which is 1 order of magnitude larger than the sheet density in bulk samples which varies between 10^10^ and 10^11^ cm^–2^.^4,13^ This exceptional density tunability allows us to map this Lifshitz transition diagram across a much wider range. It also offers the opportunity to study transport behavior across this entire density range within a single system, which is not possible for bulk systems.

The field effect mobility for holes and electrons is obtained by replotting the data of Figure 1c on a conductivity ordinate (Figure S5b, Supporting Information), using the expression^18,25^
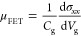
1Here, σ_*xx*_ is the sheet conductivity, *V*_g_ is the gate voltage, and  is the capacitance per unit area where *e* is the elementary charge. For our back-gate displaced from the ZrTe_5_ by a 300 nm thick SiO_2_ layer and a hBN flake, a fit to gate-dependent carrier density data from Hall measurements yields *C*_g_ = 0.970 F/cm^2^ (Section S2, SI). The mobilities are shown in Figure 1f. For both carrier types, the mobility increases gradually with decreasing temperature and saturates below ∼60 K. A hole mobility as high as ∼3500 cm^2^ V^–1^ s^–1^ is achieved at low temperature, whereas the electron mobility is much smaller (∼1000 cm^2^ V^–1^ s^–1^). This suggests an asymmetry in the band structure.^10,16^

Although these mobilities are far away from those obtained on bulk ZrTe_5_ (∼10^5^ cm^2^V^–1^s^–1^),^4,13^ QHE features at integer Landau level filling ν can be observed (Figure 2). In earlier thin film studies such QHE behavior was absent,^18−21^ likely as a result of surface degradation causing heavy hole doping and multiband transport. For well-developed quantum Hall states, the Hall conductivity σ_*xy*_ is quantized in units of  and the longitudinal conductivity σ_*xx*_ vanishes, where *h* is Planck’s constant. Figure 2a illustrates ρ_*xx*_ and ρ_*xy*_ as a function of *V*_g_, that is, density, at 13.8 T and 1.7 K. Panel Figure 2d shows both quantities as a function of *B* and fixed density for 9 K to avoid clutter from conductance fluctuations that appear at lower temperature. Incipient quantum Hall effect features are clearly visible. They are brought out even better, when performing the tensor inversion and plotting the longitudinal (σ_*xx*_ = ρ_*xx*_/(ρ_*xx*_ρ_*yy*_ + ρ_*xy*_^2^)) and Hall conductivity (σ_*xy*_ = −ρ_*xy*_/(ρ_*xx*_ρ_*yy*_ + ρ_*xy*_^2^)) instead (Figure 2b). Note that in principal the in-plane anisotropy^3,20^ (α = ρ_*yy*_/ρ_*xx*_) should be taken into consideration when calculating the conductivity tensor elements for ZrTe_5_. Even if so, such an anisotropy correction has no influence on the main conclusion (Section 14, SI). Figure 2b has been generated for α = 1. At filling ν = 1, σ_*xy*_ reaches ∼1.068 *e*^2^/*h*, close to the fully quantized value of *e*^2^/*h*. The plateau is also accompanied by a deep minimum in σ_*xx*_ (1.67 × 10^–7^ S ∼ 0.043 *e*^2^/*h*). At filling ν = 6, σ_*xx*_ also exhibits a strong minimum and at σ_*xy*_ a plateau starts to develop. Weaker QHE features are available at ν = 2 and 4. This QHE is distinct from the previously reported 3D QHE on bulk ZrTe_5_ as the latter Hall effect requires a gap opening in the third direction believed to be mediated by a transition to a charge density wave state.^2,4^ Because the effect occurs in the bulk, it is far less vulnerable compared to the reduced dimensionality needed here.

**Figure 2:**
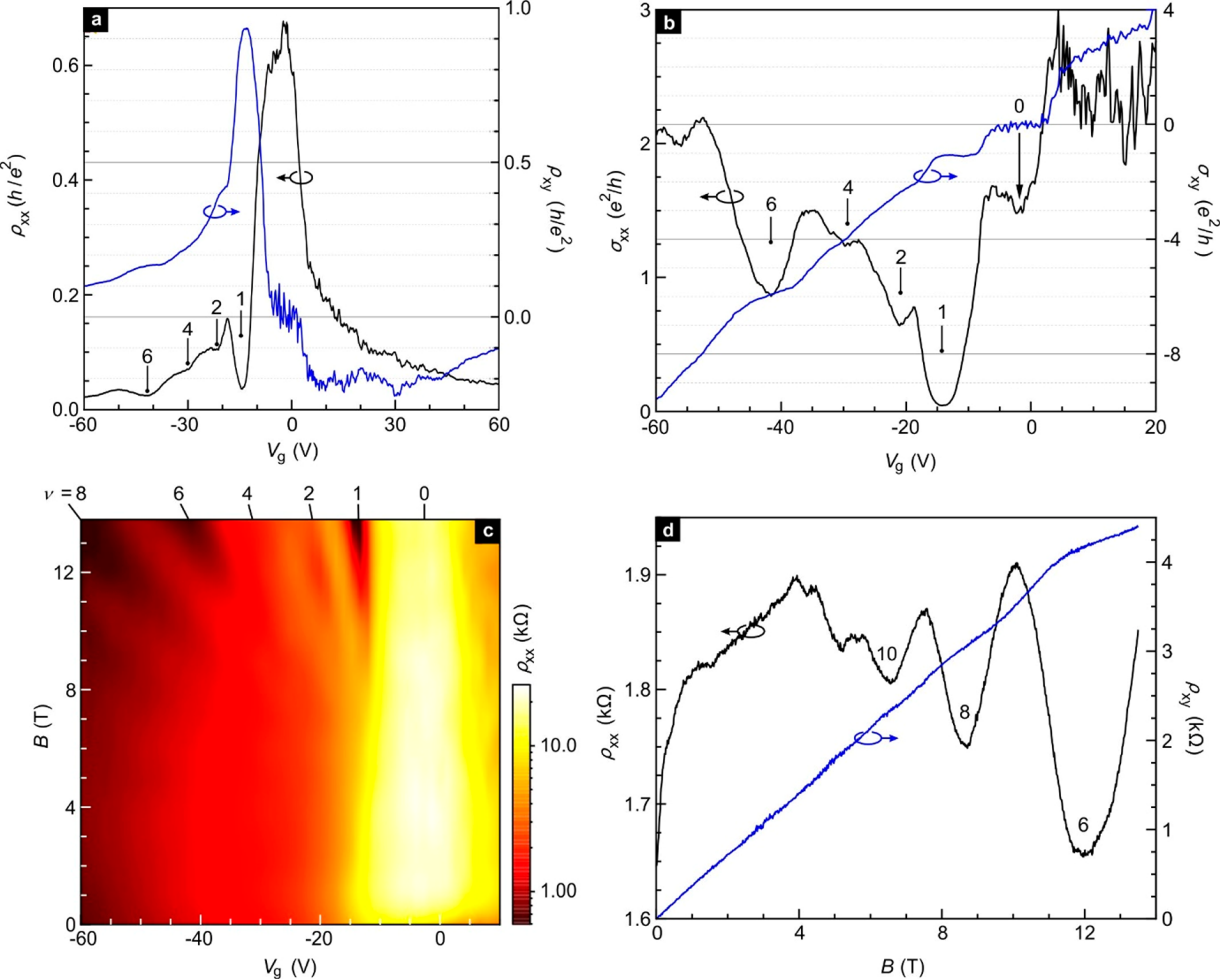
Incipient quantum Hall effect features. (a) ρ_*xx*_ (black) and ρ_*xy*_ (blue) measured as a function of *V*_g_ at a fixed *B* = 13.8 T and a temperature *T* of 1.7 K. The filling factor corresponding to each minimum is included. (b) σ_*xx*_ (black) and σ_*xy*_ (blue) obtained from (a) using the tensor inversion. (c) Color rendition of ρ_*xx*_ across the plane spanned by back gate voltage *V*_g_ and field *B.* (d) Magnetic field dependence of ρ_*xx*_ and ρ_*xy*_ for fixed *V*_g_ = −35 V at *T* = 9 K.

Particularly interesting is the plateau in σ_*xy*_ centered around zero filling, while at the same time ρ_*xx*_ has increased significantly. These are the signatures of a true insulating ground state. A color map of ρ_*xx*_ across the (*B*,*V*_g_)-plane is displayed in Figure 2c. At high fields, it resembles a Landau fan chart with dark areas signaling incompressible behavior at the fillings marked at the top. At low fields, the insulating phase centered around ν = 0 dominates. Examples of the *B*-dependence of ρ_*xx*_ and ρ_*xy*_ at a fixed back gate voltage of *V*_g_ = −35 V are illustrated in Figure 2d. Minima are observed at even fillings ν = 6, 8, and 10 consistent with 2-fold spin degeneracy of the single Dirac-conelike dispersion at the Γ-point in reciprocal space.^4,13,16^ Lower temperature is required to resolve the field-induced Zeeman splitting due to substantial disorder broadening (Section S9, SI). We note that the QHE in Figure 2a,b only appears for hole doping where the mobility is highest. For electrons with their lower mobility, the data reveal fluctuations as observed previously in exfoliated few-layer quasi-one-dimensional thin films.^18−21^ They may be the result of additional scattering. For thicker samples where surface inhomogeneities play a lesser role, Shubnikov–de Haas (SdH) oscillations can also be observed for electrons (see Figure S8).

A more detailed analysis of the Shubnikov–de Haas oscillations allows the extraction of important band structure parameters. The amplitude of the 1/*B*-periodic oscillations can be described by the Lifshitz–Kosevich formula^4,13^
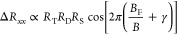
2Here, *R*_T_, *R*_D_, and *R*_S_ are damping factors accounting for phase smearing due to temperature, disorder scattering, and spin splitting, respectively. The maximal Fermi surface section perpendicular to the *B*-field is denoted as *B*_F_ and γ is the Berry phase.^4,13,24^Figure 3a displays the Shubnikov–de Haas oscillation amplitude Δ*R*_*xx*_ at a fixed gate voltage of *V*_g_ = −35 V as a function of 1/*B* for different temperatures. A smooth background was subtracted from the raw magneto-resistance data to obtain Δ*R*_*xx*_ and improve the visibility of the oscillations. Arrows mark integer fillings where minima are expected. Minima due to the Zeeman splitting are resolved only in traces recorded at the lowest temperatures. A fast Fourier transform of  recorded at 9 K has been included as an inset in Figure 3a. A single frequency of ∼29 T is resolved. This confirms single band transport. The temperature dependence of the amplitude can be described with damping factor . The parameter λ(*T*) equals , where *k*_B_ is the Boltzmann constant, *ℏ* is the reduced Planck constant, and the cyclotron mass m* is a geometrical average of the effective masses in the plane. Some fits of the *T*-dependence of  at different *B*-fields are illustrated in Figure 3b for *V*_g_ = −35 V. They yield a hole cyclotron mass m* of about 0.085 ± 0.01 *m*_e_, where *m*_e_ is the free electron mass. This cyclotron mass is significantly larger than the value of ∼0.02 *m*_e_ reported for bulk.^4,13^Figure 3b plots m* extracted from the *T*-dependent analysis of the Shubnikov–de Haas oscillations for different densities revealing a strong dependence. The effective mass is highest for large hole density and gradually drops with decreasing density. Scattering is also responsible for a *B*-dependent damping of the Shubnikov–de Haas oscillations. It is taken care of in eq 2 by the factor , where *D* is equal to . By fitting the field dependence in a Dingle plot showing ln[Δ*R*_*xx*_*B* sinh λ(*T*)] as a function of 1/*B* the quantum lifetime τ_q_ can be determined (insert to Figure 3c). For different hole doping, we obtain a τ_q_ up to 80 fs (Figure 3c).

**Figure 3:**
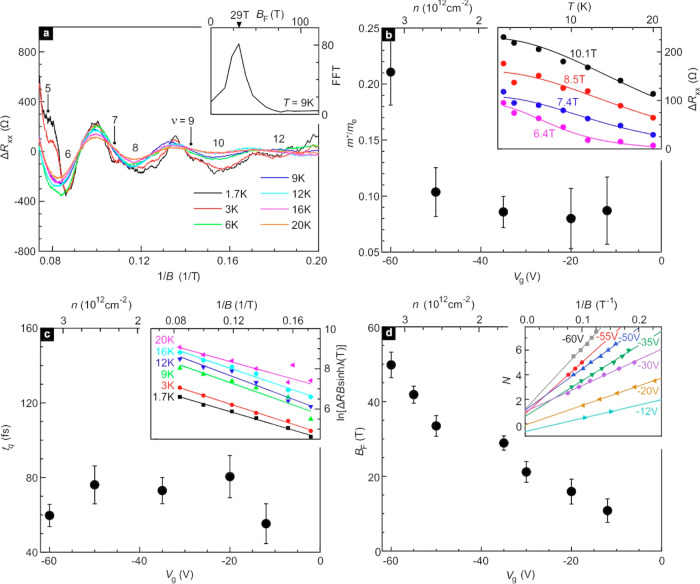
Analysis of the Shubnikov–de Haas oscillations. (a)  as a function of 1/*B* at different temperatures and fixed *V*_g_ = −35 V. Numbers are integer filling factors where minima are expected. The inset displays the fast Fourier transform of the trace recorded at *T* = 9 K. (b) Hole cyclotron mass at different gate voltages, that is, carrier densities. The mass is obtained from fits to the temperature-dependence of the oscillation amplitude as shown for *V*_g_ = −35 V in the inset at different magnetic fields. (c) Hole carrier quantum lifetime for different gate voltages, that is, carrier densities. These lifetimes are obtained from Dingle plot fits. The inset example shows such Dingle plots for *V*_g_ = −35 V at different temperatures. (d) *B*_F_ at different gate voltage, obtained from the Landau diagrams as shown in the inset.

By assigning an integer Landau level index *N* to each maximum of the oscillation amplitude , that is, *N* = , it is possible to extract *B*_F_ as the slope in a Landau index graph plotting *N* versus 1/*B* as shown in the inset to Figure 3d. The carrier density *n*_SdH_ participating in the SdH oscillations is obtained from  and was also calculated and compared with Hall measurements in Figure S2b. The intercept of the linear fit to the maxima in the inset of Figure 3d should yield the Berry phase. It can take on values between 0 and 1 and can provide evidence for topological properties of the bands. Unfortunately, no meaningful information can be extracted here, which we primarily attribute to the moderate quality of the oscillations (Section 10, SI). The large Zeeman splitting further complicates the picture as it can distort the phase of the oscillations. In bulk systems,^4,13^ low magnetic field data (usually less than 2 T) were chosen to avoid the Zeeman splitting related phase shift in the oscillations. It is possible to estimate the *g*-factor through the third damping factor  in eq 2. From a fit in the Landau index plot for each spin ladder,^13^ (see SI, Figure S10b), a *g*-factor of 11.2 ± 2.8 is obtained. For bulk ZrTe_5_, this *g*-factor can even be as large as 15.8 or 21.3.^13,14^ An estimate of the ratio of the cyclotron energy () and the Zeeman energy () yields a value of 2.1 ± 0.4. Angular-dependent data would be necessary for a proper quantitative analysis of the *g*-factor. However, this is beyond the capabilities of our vacuum sealed probe technique.

Finally, we turn our attention to the ν = 0 state observed near the charge neutrality point in Figure 2. In Figure 4a, σ_*xy*_ is plotted as a function of gate voltage for different *B-*fields. Curves are shifted by *e*^2^/*h* for ease of comparison. Features associated with filling 1, 2, 4, and 6 are marked and remind of the Landau level map discussed in Figure 2c. Plateaus or incipient plateau features separate and span a larger range of *V*_g_ with increasing *B-*field due to the increase in the Landau level degeneracy and cyclotron energy. However, for the ν = 0 state there is no obvious magnetic field dependence above 1 T. The black dashed lines in Figure 4a are guides to the eye that attempt to demarcate the width of this ν = 0 state. The width remains more or less constant with increasing field. In graphene^26^ and HgTe quantum wells,^27^ the ν = 0 state appears when the degeneracy of the *N* = 0 Landau level at zero energy is completely lifted and an insulating state is induced by the magnetic field. However, in both systems the width of the ν = 0 plateau increases monotonically with magnetic field. This is different from our observations here on the ZrTe_5_ system. As shown in Section S11 in the SI, the ν = 0 plateau is also very robust against thermal activation. It survives beyond 100 K suggesting that neither the cyclotron energy gap nor the Zeeman splitting are responsible but instead an intrinsic bulk band gap is responsible. The latter is crucial for the realization of the quantum spin Hall effect.

**Figure 4:**
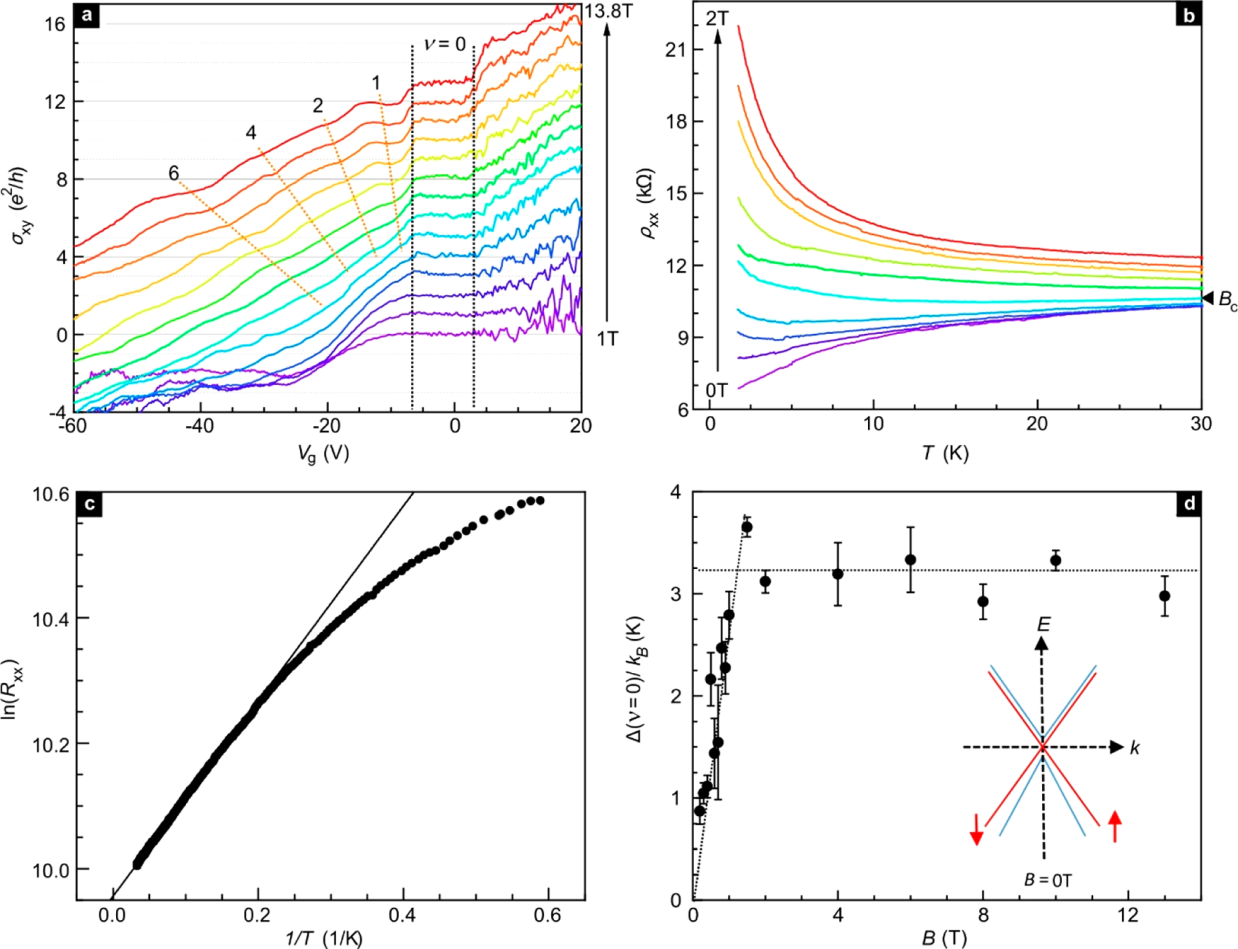
Insulating ν = 0 ground state near the charge neutrality point. (a) σ_*xy*_ as a function of gate voltage for different magnetic fields. The data are recorded at *T* = 1.7 K. All curves are shifted vertically *e*^2^/*h* for clarity. Integers and dashed lines mark the filling factor of the plateau and incipient plateau features in σ_*xy*_. The dashed lines are guides to the eye demarcating the ν = 0 state. (b) Metal-to-insulator transition at ν = 0 state with increasing magnetic field. The critical field *B*_c_ is approximately equal to 0.2 T. (c) Arrhenius plot showing ln(*R*_*xx*_) as a function of 1/*T* to estimate the activation energy gap at ν = 0 state. (d) Energy gap obtained from Arrhenius plots at different magnetic fields. The gap is fully developed and remains constant above ∼2 T. The inset shows a schematic of the band structure for few-layer ZrTe_5_ that would be compatible with the experimental observations.

To determine the gap size, the temperature-dependence of ρ_*xx*_ is studied for the ν = 0 state at different *B*-fields. Figure 4b covers data for fields below 2T, and complementary data at higher fields are shown in Section S12, SI. A metal–insulator transition at a critical field *B*_c_ of approximately 0.2 T can be observed. Above 2 T, curves fall on top of each other indicating a nearly field independent gap. It is estimated from an Arrhenius plot^26,28^ of  (Figure 4c). The gap value is plotted in Figure 4d. A gap appears around 0.2 T. Its value increases rapidly up to 2 T and then saturates at higher field. Its high field value is approximately equal to 3.2 ± 0.4 K (∼0.28 meV). These experimental observations are compatible with ZrTe_5_ being either a weak or strong TI. Since the interlayer spacing is about 7.25, the sample consists of as many as 9 ± 1 layers, so the thin film is presumably best described as 3D. In 3D, both a weak and strong topological insulator possesses an intrinsic bulk gap. For a strong TI, all surfaces have gapless states, whereas for a weak TI the top and bottom surfaces are in general gapped; however, the four perpendicular surfaces have gapless states as well.^29^ Hence, irrespective of a weak or strong topological insulator, we end up with a band diagram schematically shown in the inset to Figure 4d consisting of a bulk band with a gap as well as gapless surface or edge states that are spin polarized due to spin-momentum locking.^30^ If the chemical potential is adjusted to lie inside the gap of the bulk band, the gapless surface states support metallic behavior at low fields (*B* < *B*_c_). These topological surface states, responsible for the quantum spin Hall state, are however easily destroyed when time reversal symmetry gets broken through the application of a large enough *B-*field. The sample is then expected to turn into an insulator. If instead we are dealing with a 3D Dirac semimetal, then there is no bulk gap and we would not expect to observe the *v* = 0 ground state.

The gap value we report here is much smaller than previously reported values. The first theoretical work predicted a quantum spin Hall gap as large as 100 meV in monolayer ZrTe_5_.^3^ ARPES and STM measurements performed on the surface of ZrTe_5_ bulk material confirmed a bulk gap with a value of 80–100 meV.^10,11^ However, subsequent theoretical work as well as high-resolution spectroscopy studies suggested that the energy gap is strongly temperature dependent with a maximum of about 30 meV.^16,17,31,32^ Recent optical spectroscopy measurements even obtained a gap value as small as 6 meV.^33^ These discrepancies between different reports raise controversy about the actual gap value. Part of the problem is no doubt that experimental studies performed on actual monolayers or ultrathin films are lacking. Despite these discrepancies, our work confirms the existence of a band gap in magneto-transport. Taken altogether our experimental observations seem to exclude that the ZrTe_5_ sample is a 3D Dirac semimetal, while it can be either a weak or strong topological insulator. Further progress in sample quality will likely turn thin ZrTe_5_-based field effect devices into a useful platform to investigate topological phase transitions and search for the quantum spin Hall state in this highly tunable system.

## Supporting Information Available

Sample thickness determination, carrier density analysis, zero field resistance map, electron hole transition, field effect mobility analysis, QHE and SdH oscillations with different thickness, SdH analysis, Berry phase and g-factor, *v* = 0 state evolution, Arrhenius analysis of the energy gap, and conductivity tensor correction (PDF)
