# Supplementary material for: Two-Dimensional Quantum Hall Effect and Zero Energy State in Few-Layer ZrTe5
Source: Nano Lett. 2021 Jul 12;21(14):5998–6004. doi: 10.1021/acs.nanolett.1c00958 (PMC8397394; doi:10.1021/acs.nanolett.1c00958)
Supplement: Supplementary file 1 — nl1c00958_si_001.pdf [file nl1c00958_si_001.pdf]

Supporting Information

Two-dimensional quantum Hall effect and zero energy state in few-layer $ZrTe_5$

Fangdong Tang[1], Peipei Wang[2], Mingquan He[3], Masahiko Isobe[1], Genda Gu[4], Qiang Li[4,5], Liyuan Zhang[2], Jurgen H. Smet[1]

[1] Max Planck Institute for Solid State Research, Stuttgart 70569, Germany

[2] Department of Physics and Shenzhen Institute for Quantum Science and Engineering, Southern University of Science and Technology, Shenzhen 518055, China

[3] Low Temperature Physics Laboratory, College of Physics & Center of Quantum Materials and Devices, Chongqing University, Chongqing 401331, China

[4] Condensed Matter Physics & Materials Science Division, Brookhaven National Laboratory, Upton, New York 11973-5000, USA.

[5] Department of Physics and Astronomy, Stony Brook University, Stony Brook, NY 11794-3800, USA

Content

1. Sample thickness determined by AFM

2. Back-gate operation and carrier density

3. Zero field resistance map in the (V_g, T) plane for samples of different thickness

4. Transition from electron doping to hole doping

5. Field effect mobility

6. Changes in sample quality upon exposure to air or elevated temperature

7. Emergence of the QHE and SdH oscillations with decreasing $ZrTe_5$ layer thickness

8. SdH oscillations in thicker samples with electron doping

9. SdH oscillations for hole doping

10. Attempt to extract the Berry phase and g-factor

11. The $\nu = 0$ state and its evolution with magnetic field and temperature

12. Arrhenius analysis for the determination of the activation energy gap of the $\nu = 0$ state

13. Arrhenius analysis for the $\nu = 0$, 1 and 2 ground states at 13 T

14. Conductivity tensor correction considering in-plane anisotropy

15. Methods

16. References

1. Sample thickness determined by AFM

In order to determine the thickness of the $ZrTe_5$ layer, atomic force microscopy (AFM) was deployed. Panel **a** of Fig. S1 shows an AFM image recorded on device D1, discussed in detail in the main text. Panel **b** is a line trace of the height across the active device area. We extract a thickness of 6.5 $\pm$ 1.1 nm on this device. The surface of thin $ZrTe_5$ is rather rough. This observation is in agreement with what has been reported previously[1,2]. Increased surface roughness is accompanied by a drop in the sample mobility. The more homogeneous the sample thickness, the more pronounced quantum oscillations are.

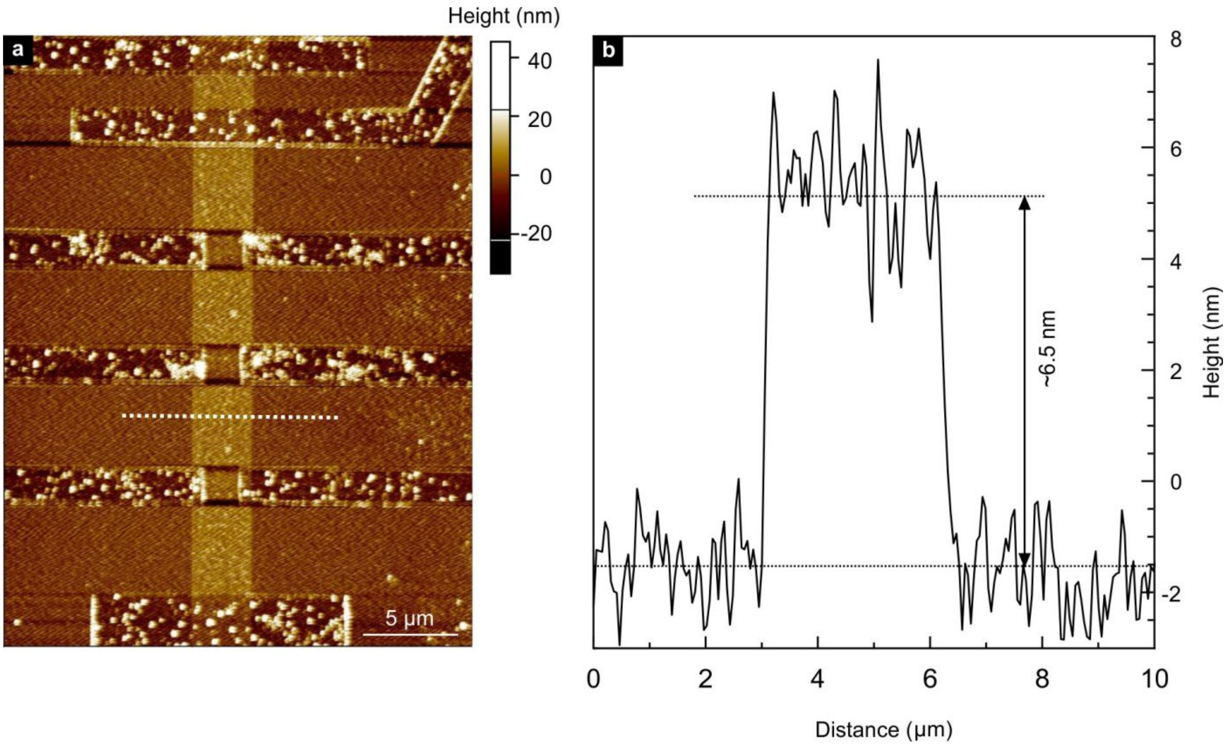

Fig. S1 | Sample thickness determined from atomic force microscopy. a, AFM image recorded on device D1. **b.** Line trace of the recorded height along the dashed line in panel **a.**

2. Back-gate operation and carrier density

In order to extract the sample carrier density, we either use a linear fit to the recorded Hall resistivity with the expression $R_{\mathrm{H}} = \frac{1}{n_{\mathrm{Hall}} e}$ or determine the periodicity of the Shubnikov-de Haas oscillations in the longitudinal resistance (see fan diagram of the oscillation minima in Fig. 3d of the main text), $n_{\mathrm{SdH}} = \frac{2eB_{\mathrm{F}}}{h}$. Panel **a** of Fig. S2 shows an example of the Hall resistivity and the corresponding fit. In panel **b** both Hall density, n_{Hall}, and Shubnikov-de Haas density, n_{SdH}, are plotted as a function of the back-gate voltage applied to the doped Si substrate. Since the uncertainty of the Hall density is smallest, it is used here to determine the capacitance in the field effect model from a linear fit to the data. This yields a capacitance of $C_{\mathrm{g}} = 0.970$ F/cm^2 for this particular device. The scatter in the data on the electron side is much larger and therefore not considered in the analysis. At higher field effect, the hole doping from SdH oscillations, n_{SdH}, deviates from the Hall density data and the linear fit. It is systematically smaller compared with n_{Hall}. This suggests the co-existence of low-mobility residual carriers that do not participate in the quantum oscillations.

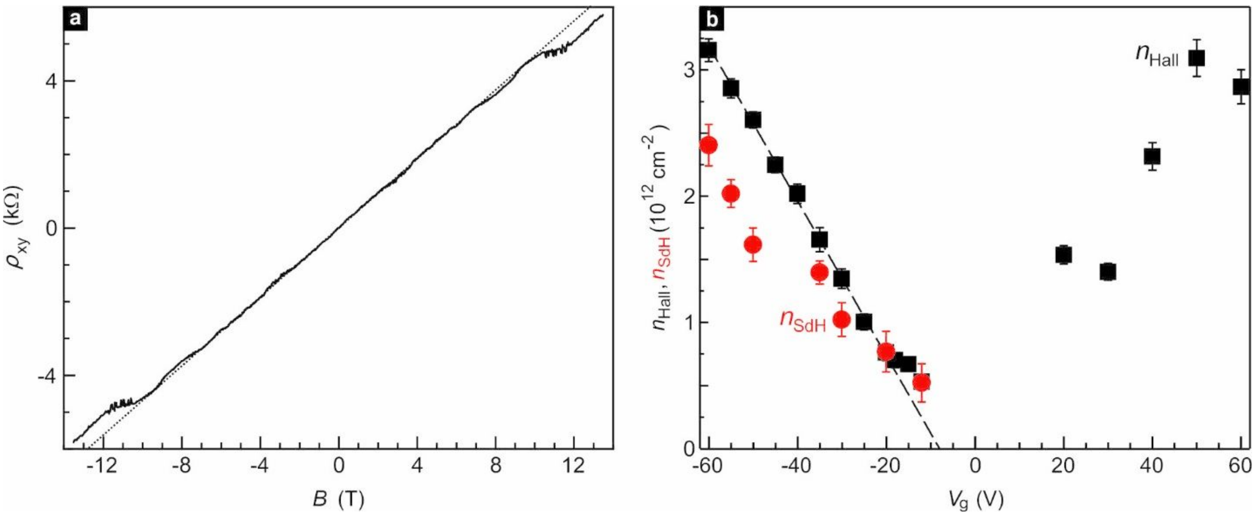

Fig. S2 | Carrier densities extracted from Hall and longitudinal resistance measurements and their dependence on the back-gate voltage V_{g}. a, Example of the Hall resistivity recorded on device D1 at V_{g} = -30 V and T = 1.7 K in the low magnetic field range (2 T). The Hall coefficient and Hall density are obtained from a linear fit to the data (dotted line). **b,** Hall and Shubnikov-de Haas density as a function of the applied back-gate voltage. The dashed line is a linear fit to the n_{Hall}-data in order to extract the gate capacitance.

3. Zero field resistance map in the (V_g, T) plane for samples of different thickness

Two examples of the resistance behavior as a function of back-gate voltage and temperature in the absence of an applied magnetic field are shown in Fig. S3. The left panel is for the 9.5 $\pm$ 1.5 nm thick $ZrTe_5$ layer referred to as device D3, while the right panel plots the data for device D5 with a $ZrTe_5$ layer thickness of 13 $\pm$ 3 nm. Similar behavior is observed for all other devices that exhibit a clear maximum in the resistance as a function of temperature for the case of electron doping.

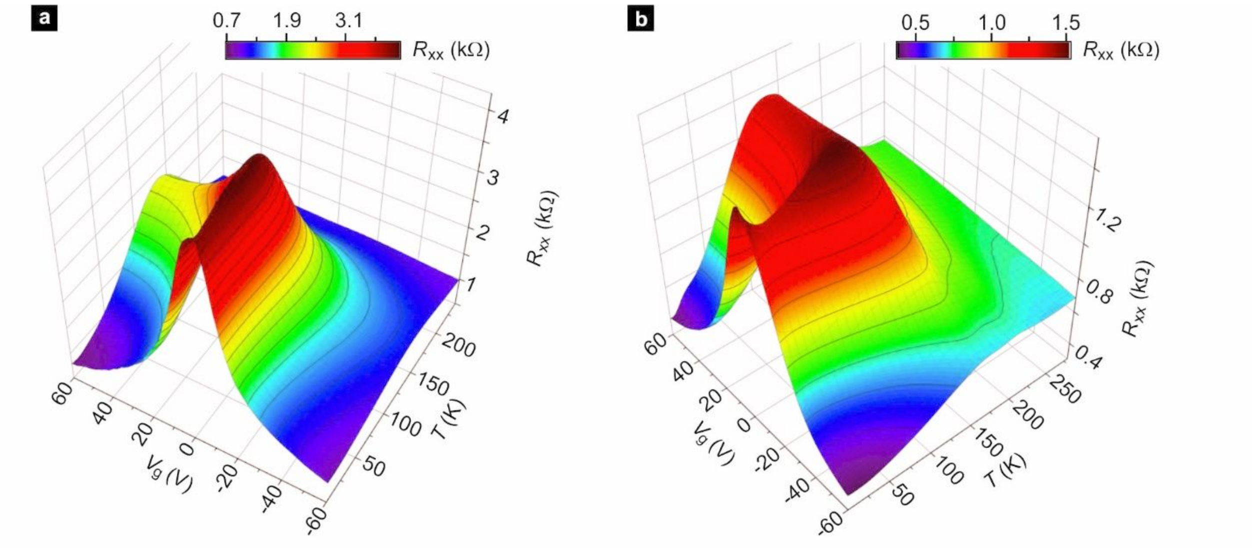

Fig. S3 | 3D plot of the resistance across the (V_g, T) plane at zero magnetic field for samples of different thickness. **a**, Data for device D3 with a 9.5 $\pm$ 1.5 nm thick $ZrTe_5$ layer. **b**, Data for device D5 with a 13 $\pm$ 3 nm thick $ZrTe_5$ layer.

4. Transition from electron doping to hole doping

In order to determine the transition from electron doping to hole doping, three distinct procedures can be used, each of which is illustrated in the panels of Fig. S4 using data acquired on device D1. The first approach is based on the anomalous maximum that forms in the temperature dependence of the longitudinal resistance at a fixed back-gate voltage (panel a). Alternatively, the transition point at a given temperature can be obtained from the zero crossing of the Hall resistance plotted for a fixed magnetic field (here 1 T and -1 T) as a function of the back-gate voltage (panel b), i.e. $\rho_{xy}(V_g) = 0$. In order to remove the undesirable contribution

of the longitudinal resistance to the measured resistance, the Hall resistance trace is symmetrized using the expression

$$\rho_{xy}(V_g, 1\text{T})_{\text{sym}} = \frac{\rho_{xy}(V_g, 1\text{T}) - \rho_{xy}(V_g, -1\text{T})}{2}.$$

Finally, also field effect traces recorded at fixed temperature allow the extraction of the transition point by determining the peak position, i.e. $\frac{d\rho_{xx}}{dV_g} = 0$.

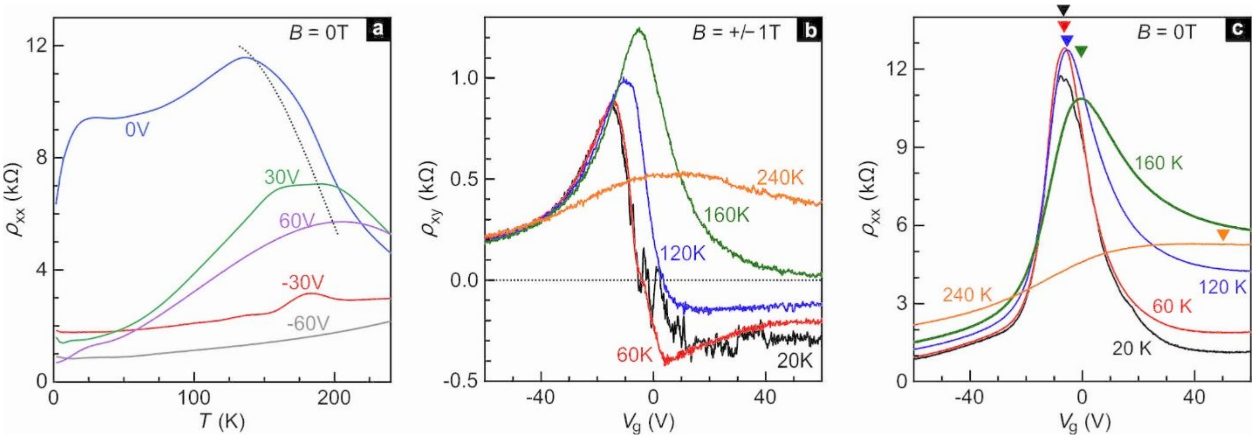

Fig. S4 | Three distinct ways to determine the transition from hole doping to electron doping. All data shown were recorded on device D1. **a**, Longitudinal resistivity as a function of temperature for different back-gate voltages. The anomalous resistivity maximum in traces obtained at back-gate voltages that generate electron doping at low temperature marks the transition from hole to electron doping. The dotted line connects these identified maxima related to the transition. **b**, Symmetrized Hall resistivity as a function of the gate voltage obtained for a fixed magnetic field of +1 and -1 T. The crossing through zero marks the transition between electron and hole doping. **c**, Field effect curves of the longitudinal resistivity recorded at a fixed temperature. Maxima ($d\rho_{xx}/dV_g = 0$) signal the transition between electron and hole doping and are marked by triangles.

5. Field effect mobility

Field effect curves recorded at zero magnetic field for different sample temperatures are plotted in Fig. S5a. For temperatures below 120 K pronounced peaks are visible. They remind of the

so-called Dirac peak in graphene samples. At temperatures beyond 200 K, the maximum has disappeared indicating that the chemical potential has moved deep into the valence band and it is no longer possible to enter the electron doped regime. In panel b some of these traces have been replotted using conductivity rather than resistivity as the ordinate. From the slope of a linear fit to these traces in the regime of low hole density the field effect mobility μ_{FET} is estimated as illustrated by the dotted lines. These mobilities for holes as well as electrons from similar fits are summarized in Fig. 1f of the main text.

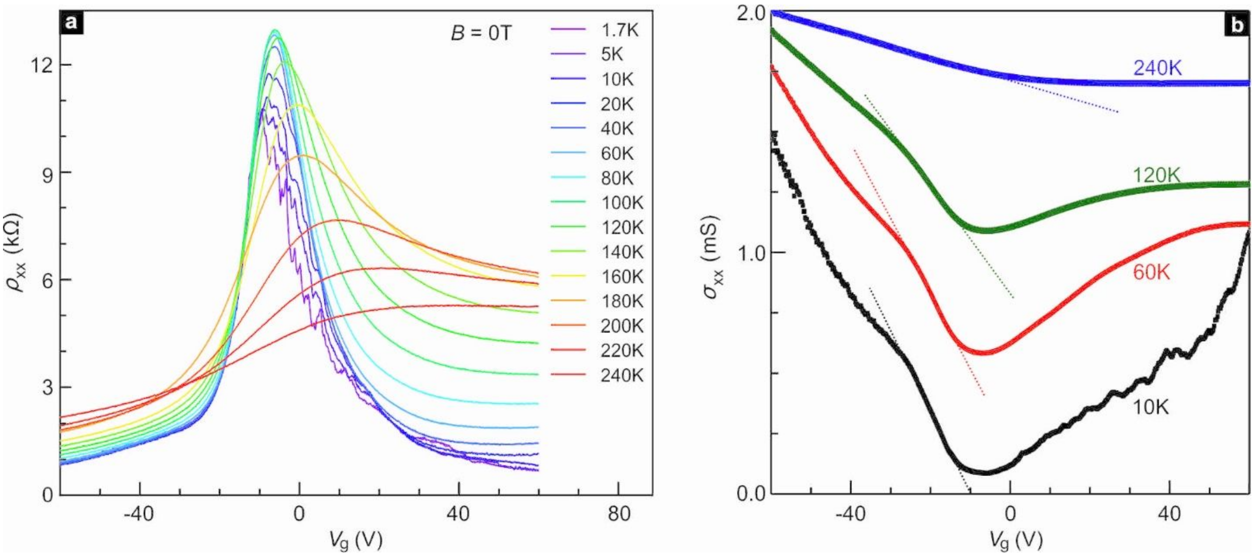

Fig. S5 | Illustration of field effect curves used for the extraction of the field effect mobility at different temperatures. All data were obtained on device D1. **a**, Field effect curves of the longitudinal resistivity measured in the absence of a magnetic field for different temperatures. **b**, Replot of selected curves of panel a on a conductivity ordinate. Dotted lines are linear fits to the data in the regime of low electron density. Their slopes yield the field effect mobility μ_{FET}.

6. Changes in sample quality upon exposure to air or elevated temperature

$ZrTe_5$ layers are both sensitive to air as well as elevated temperature treatments. Fig. S6 illustrates this sample degradation on device D3 and D4. Panel a compares the longitudinal resistance after an additional exposure to 1 and 2 hours of ambient atmosphere. For this device (D3), the $ZrTe_5$ layer has an estimated thickness of 9.5 ± 1.5 nm and is covered by an hBN layer. Panel a plots the longitudinal resistance as a function of temperature. The anomalous resistance peak associated with the transition from hole doping to electron doping moves to

higher temperature and the resistance drops significantly for longer exposures to air. This behavior suggests increased hole doping upon air exposure. In panel b, the longitudinal resistance R_{xx} (left) and Hall resistance R_{xy} (right) are plotted as a function of gate voltage at a fixed field of 13 T. These data sets corroborate that air exposure indeed causes additional hole doping. This air-induced hole doping has also been discussed in Ref. 1 and 2. The hBN cap layer is apparently not able to protect against air degradation. In AFM measurements, typically bubbles are observed between the hBN and $ZrTe_5$, which possibly accounts for the ineffectiveness of the hBN to prevent air degradation. In Panel c and d, the same experiments were repeated on device D4, but instead of exposing the sample to air it was mildly annealed at a temperature of 380 K. The anneal was performed in-situ, i.e. inside the Dynacool Physical Properties Measurement System with an accessible temperature range of 1.7 K to 400 K and with helium exchange gas in the variable temperature insert. The temperature treatment causes electron doping instead. It is also harmful for the quantum Hall effect. Previous studies on CVT and flux-grown samples[3,4] indicated that Te atom vacancies are responsible for electron doping and hence a thermal anneal may indeed be accompanied by the loss of Te.

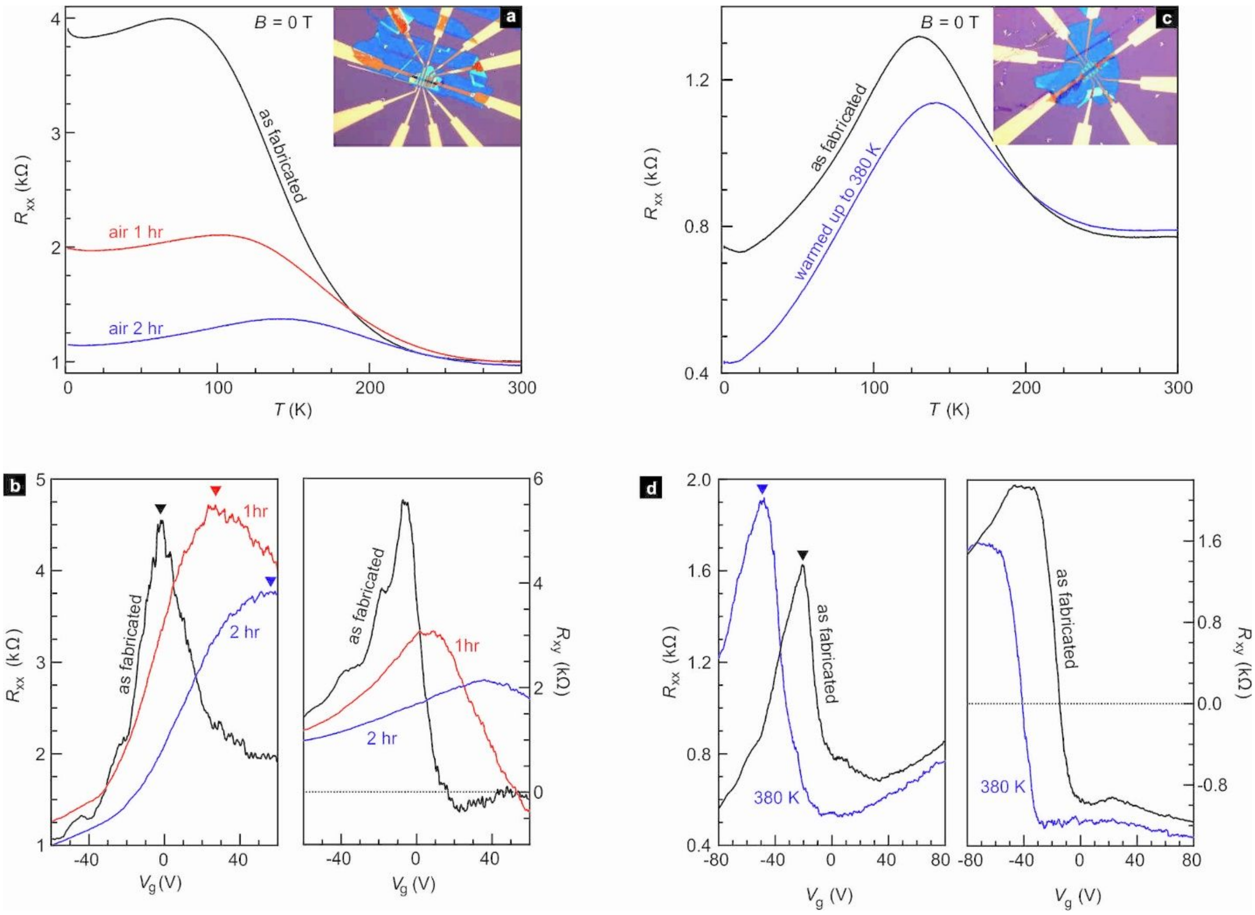

Fig. S6 | Influence of air exposure and temperature treatment on the sample properties and quality. a, Comparison of the temperature dependent longitudinal resistance recorded on device D3 upon an additional 1 or 2 hour exposure to ambient atmosphere with the behavior prior to this exposure. **b,** Comparison of the longitudinal resistance and Hall resistance recorded at 1.7 K and 13 T as a function of gate voltage when the device is exposed to ambient atmosphere for an additional 1 or 2 hours and prior to such an additional exposure. **c,** Comparison of the temperature dependent longitudinal resistance prior to and after device D4 was heated up to $T = 380$ K with helium exchange gas in a variable temperature cryostat for about 30 minutes. **d**, Comparison of the longitudinal resistance R_{xx} (left) and Hall resistance R_{xy} (right) as a function of gate voltage for device D4 prior to and after heating up the sample to temperature $T = 380$ K. The measurements were performed at 1.7 K and 13 T.

7. Emergence of the QHE and SdH oscillations with decreasing $ZrTe_5$ layer thickness

Shubnikov-de Haas oscillations and the quantum Hall effect gradually appear when successively reducing the $ZrTe_5$ thickness, provided the material quality can be preserved. Fig. S7 illustrates clearly this trend on four devices with $ZrTe_5$ layer thicknesses ranging from about 12 nm down to 6 nm. Shubnikov-de Haas minima are marked with dotted lines and identified by the filling factor of the Landau quantized hole system. Clearly, both the Hall quantization and the oscillation minima become more pronounced with decreasing layer thickness.

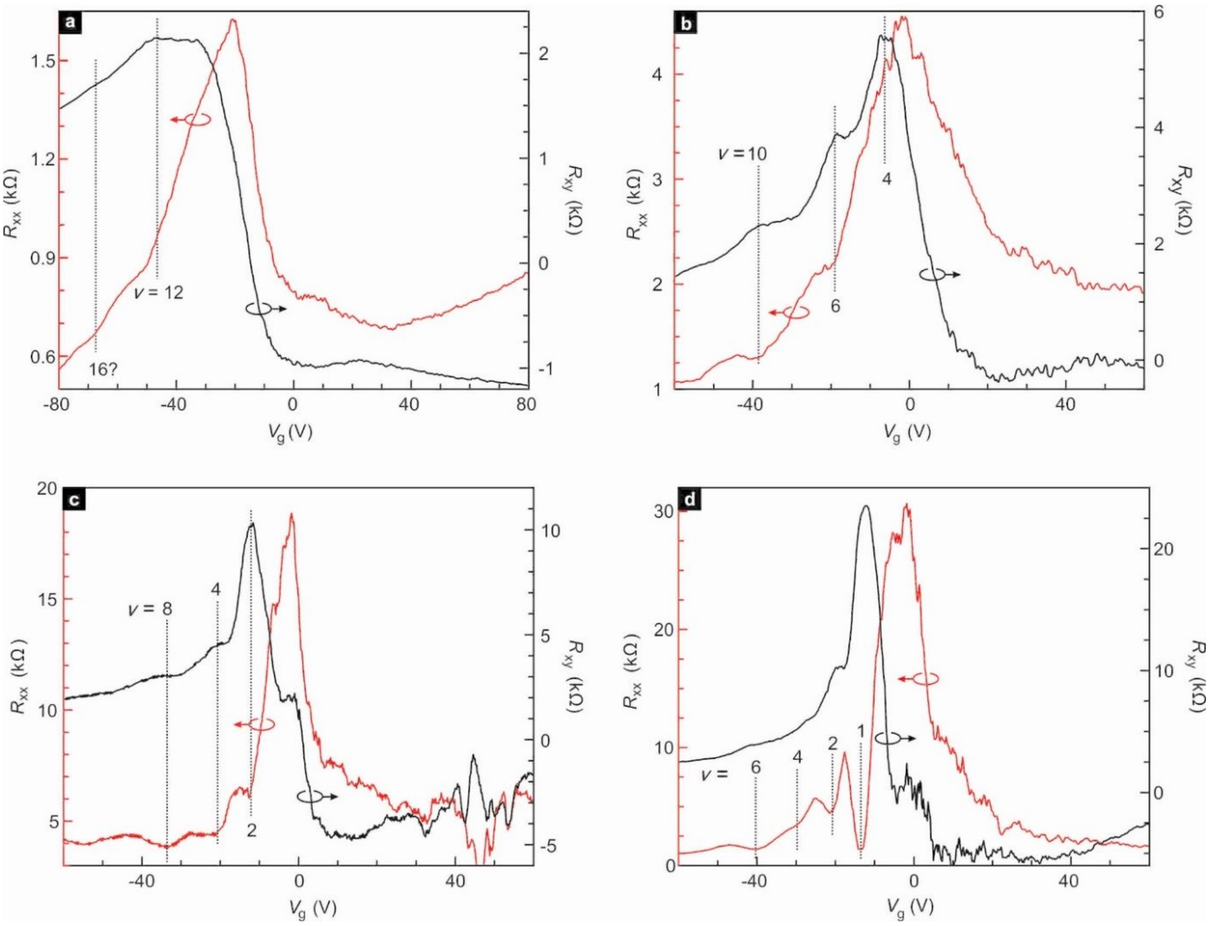

Fig. S7 | Emergence of Shubnikov-de Haas oscillations and Hall quantization as the $ZrTe_5$ layer thickness is reduced. All data are recorded at 1.7K and 13 T. **a** Longitudinal resistance R_{xx} and Hall resistance R_{xy} for device D4 with a $ZrTe_5$ layer thickness of 11.6 ± 1.8 nm, **b** Same but for device D3 with a 9.5 ± 1.5 nm thick $ZrTe_5$ layer, **c** Same but for device D2 with a 7.6 ± 1.2 nm thick $ZrTe_5$ layer. **d** Same but for device D1 with a 6.5 ± 1.1 nm thick $ZrTe_5$ layer. Dotted lines highlight Shubnikov-de Haas oscillation minima. Numbers refer to the corresponding filling factor of the hole system.

8. SdH-oscillations in thicker samples with electron doping

In devices made out of thicker $ZrTe_5$ (layer thicknesses > 10 nm), oscillations appear for large electron doping (V_g > 30 V). An example is shown in Fig. S8a for device D5 with a layer thickness of 13 $\pm$ 3 nm and a back-gate voltage of 60 V. In such samples, the Hall resistance typically varies in a non-linear fashion with B. In panel b the quantum oscillations are emphasized by using the inverse of the magnetic field as the abscissa and ΔR_{xx}, obtained by subtracting the smooth background, as the ordinate. The different curves correspond to different sample temperatures. There are no signs of Zeeman splitting in these experiments. The electron cyclotron mass is extracted from the temperature dependence of the oscillation amplitude following the procedure illustrated in panel c. The extracted mass value varies somewhat with magnetic field, but lies in the range of $m^* \sim 0.12\ m_e$, where m_e is the free electron mass. The oscillation extrema can be assigned a particular filling factor by plotting their location on the inverse magnetic field abscissa and assigning each minimum an integer value and each maximum a half integer value. The intercept of the line passing through these data points with the y-axis is non-zero ($\gamma \sim 0.468 \pm 0.1$), indicating that electrons acquire a non-trivial Berry phase of π as they complete their cyclotron orbit. The oscillation period is equal to $B_F \sim 32.5 \pm 0.7$ T, whereas the Fast Fourier Transform in the inset yields $B_F \sim 34.4$ T. Using a Dingle plot, a carrier lifetime of $\tau_q \sim 165$ fs is obtained. In thinner samples with a $ZrTe_5$ layer thickness of less than 10 nm, such well-defined quantum oscillations are typically not observed for electron doping.

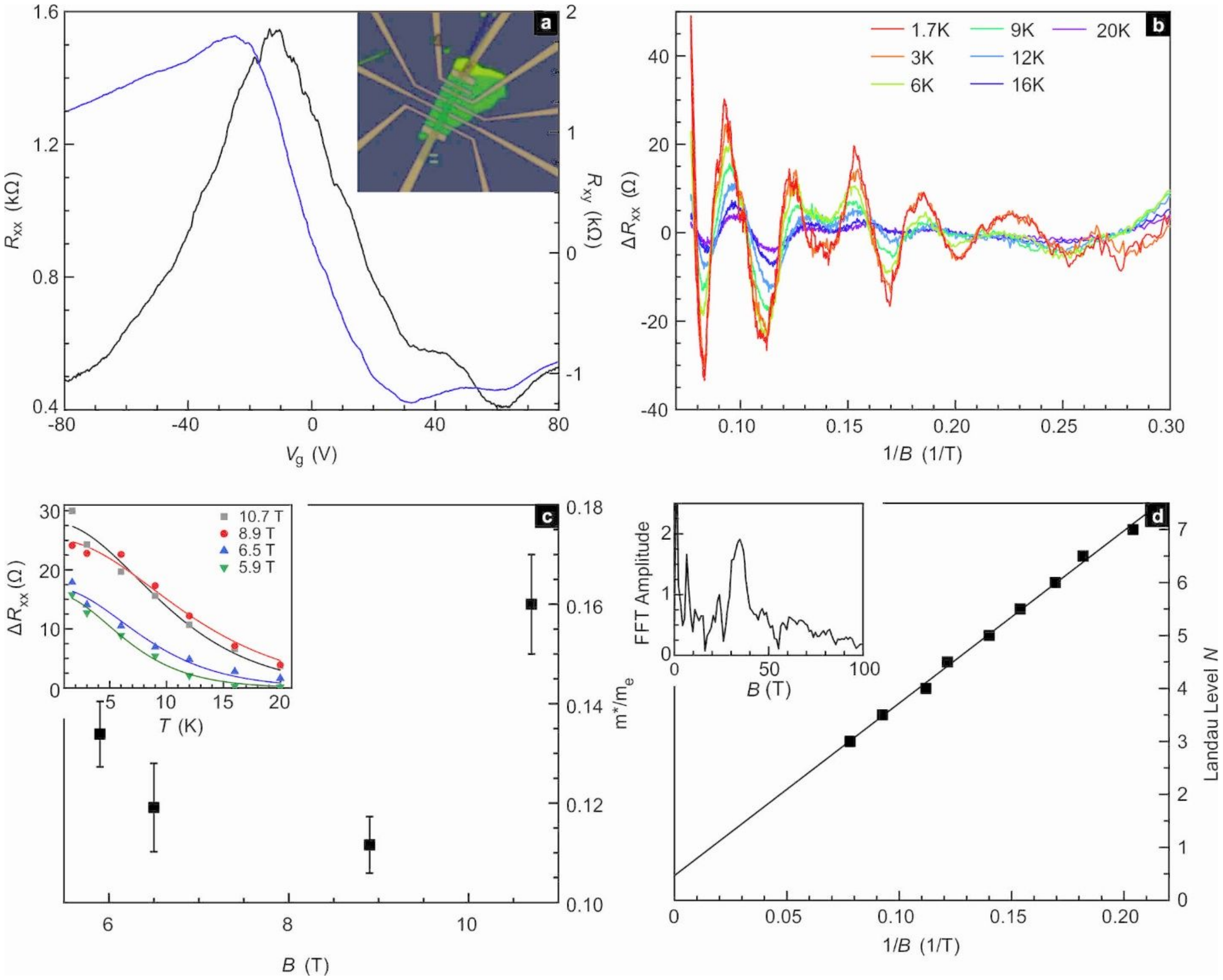

Fig. S8 | SdH oscillations for electrons in device D5 with a thicker $ZrTe_5$ layer (13 $\pm$ 3 nm). **a**, R_{xx} (black) and R_{xy} (blue) measured as a function of V_g at a fixed magnetic field of 13T and a temperature T = 1.7 K. **b**, Amplitude of the SdH-oscillations (ΔR_{xx}) as a function of $1/B$ for different sample temperatures and at a fixed gate voltage of 60 V. **c**, Electron cyclotron mass at a back-gate gate voltage V_g = 60 V obtained from fitting the temperature-dependence of the amplitude of Shubnikov-de Haas oscillations. The inset shows the fit curves for different magnetic fields, i.e. different minima. **d**, Oscillation index versus inverse magnetic field value for minima and maxima. Maxima are assigned half integers, minima integers. The inset is a Fast Fourier Transform of the data recorded at 1.7 K.

9. SdH oscillations for hole doping

Fig. S9 illustrates the temperature dependence of the longitudinal magnetoresistance R_{xx} (panel a and c) and Hall resistance R_{xy}(panel b and d) recorded on device D1 for two different hole concentrations $n_{\mathrm{Hall}} \sim 1.66 \times 10^{12}$ cm^{-2} (V_g = -35 V) (panel a and b) and $n_{\mathrm{Hall}} \sim 5.30 \times 10^{11}$ cm^{-2} (V_g = -12 V) (panel c and d). Zeeman splitting is observed at low temperatures starting from magnetic fields as small as ~ 5 T in the data acquired at V_g = -35 V (see panel a). Both disorder and thermal broadening are responsible for not resolving the spin splitting of the Landau levels at higher temperature. For this device [IS THIS CORRECT?], we extracted a g-factor of about 11. This implies a Zeeman energy of about 7.8 meV at 12T. While this is well above the thermal broadening at 9 K, the Arrhenius analysis for the ν=1 Landau level at 13T, shown in Section S13 and Fig. S14c, yields a gap size of only 1 meV. This indicates that, despite all fabrication efforts, the device still suffers from large disorder broadening (Γ_ν) of the Landau level ($\Delta E_{odd} = E_z - 2\Gamma_\nu$). In Fig. S9a, Zeeman splitting related transport features start to appear for temperatures below 6K at the lowest odd fillings. Filling factor 5 and possibly also 7 can for instance be resolved. In general, conductance fluctuations make it difficult to identify with certainty spin splitting at larger odd fillings. The ratio of the cyclotron energy ($E_c = \frac{\hbar eB}{m^*}$) and the Zeeman energy ($E_z = g\mu_B B$) was discussed in the main text and is on the order of 2. If one keeps in mind large disorder broadening, it is not surprising that features due to Zeeman splitting are much harder to resolve. The Zeeman splitting, in addition to the only moderately developed Shubnikov-de Haas oscillations, complicates an accurate determination of the Berry phase for these hole charge carriers. In the sample with lower density (panel c and d), it is possible to reach filling factor 1 for the accessible range of magnetic fields. An incipient Hall quantization is clearly observed at this filling indicating that the spin splitting in the lowest Landau level is resolved. We anticipate full quantization of this state at higher magnetic fields in conjunction with lower temperature.

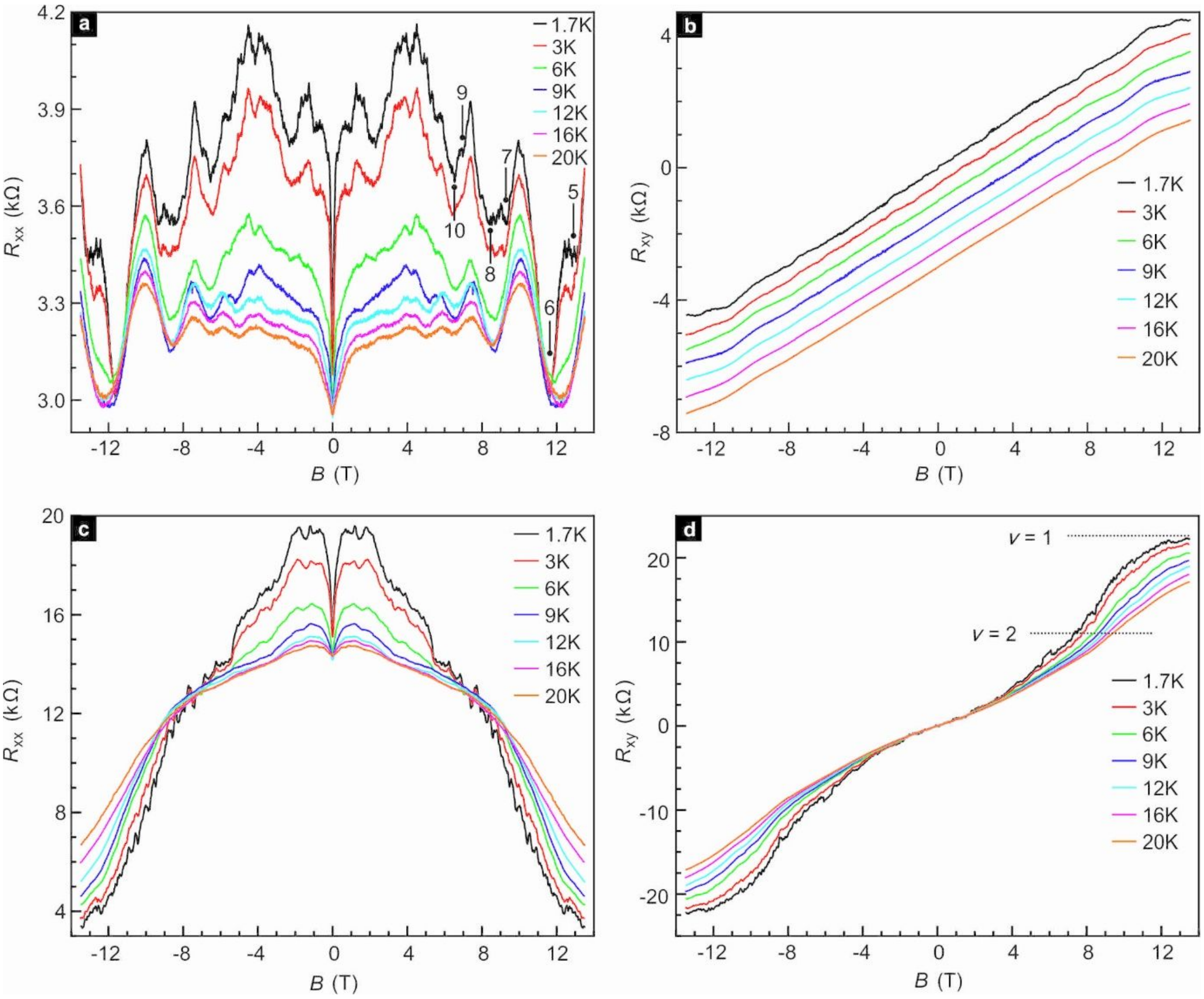

Fig. S9 | SdH oscillations obtained on device D1 for hole doping. **a**, R_{xx} as a function of magnetic field for V_g = -35 V and for the temperatures listed in the legend. **b**, Same as in panel **a**, but for the Hall resistance R_{xy}. For the sake of clarity, each curve is shifted vertically by 500 Ω. **c**, R_{xx} as a function of magnetic field for V_g = -12 V and for the temperatures listed in the legend. **d**, Same as in panel **c**, but for the Hall resistance R_{xy}.

10. Attempt to extract the Berry phase and *g*-factor

By analyzing the location of successive Shubnikov-de Haas extrema for hole doping in device D1, discussed in the main text, we have attempted to extract the Berry phase for hole charge carriers. The procedure is identical to what was discussed previously in section S8 for electron doping on the sample with the thickest $ZrTe_5$ layer (device D5, 13 $\pm$ 3 nm thick $ZrTe_5$). We used the data plotted in the inset of Fig. 3d of the main text. This analysis was repeated for multiple hole dopings and the results are summarized in panel a of Fig. S10. We conclude that no meaningful information about the Berry phase can be obtained. The Zeeman splitting is

resolved at low fields in this device causing an additional phase shift, which makes it difficult to properly and accurately identify the peaks and valleys. The main culprit remains however that, despite all efforts in improving the sample fabrication procedure, the quality of the Shubnikov-de Haas oscillations is insufficient. In panel b, the inverse magnetic field at which oscillation minima, corresponding to full occupation of the spin split Landau levels, are plotted together with their Landau level index. This exercise has been carried out in an attempt to extract the g-factor of the holes. The data points were taken for V_g = -35 V. The estimate of the g-factor is obtained using the method outlined in Ref. 5. The oscillatory component of the longitudinal magnetoresistance can be expressed as

$$\Delta R_{xx} \propto R_T R_D[\cos 2\pi\left(\frac{B_F}{B} + \gamma + \frac{1}{2}\varphi\right) + \cos 2\pi\left(\frac{B_F}{B} + \gamma - \frac{1}{2}\varphi\right)].$$

Here, γ reflects the Berry phase, $\varphi = \frac{gm^*}{2m_e}$ the phase difference between the oscillations of spin-up and spin-down holes, B_F is the period of the oscillations, and R_T and R_D are damping factors to account for the phase smearing effect of temperature and scattering. For the data plotted in panel b, this expression leads to an estimated g-factor of 11.2 ± 2.8. This large g-factor is responsible for the observability of the Zeeman splitting at low magnetic fields. An estimate of the ratio of the cyclotron energy ($E_c = \frac{\hbar eB}{m^*}$) and the Zeeman energy ($E_z = g\mu_B B$) yields a value of 2.1 ± 0.4. For the sake of completeness, we note that in the literature the following g-factors were reported for bulk $ZrTe_5$ material: 21.3 from transport data,[5] 15.8 and 22.5 from magneto-infrared spectroscopy,[6] and 40 ~ 60 from scanning tunneling microscopy (STM).[7]

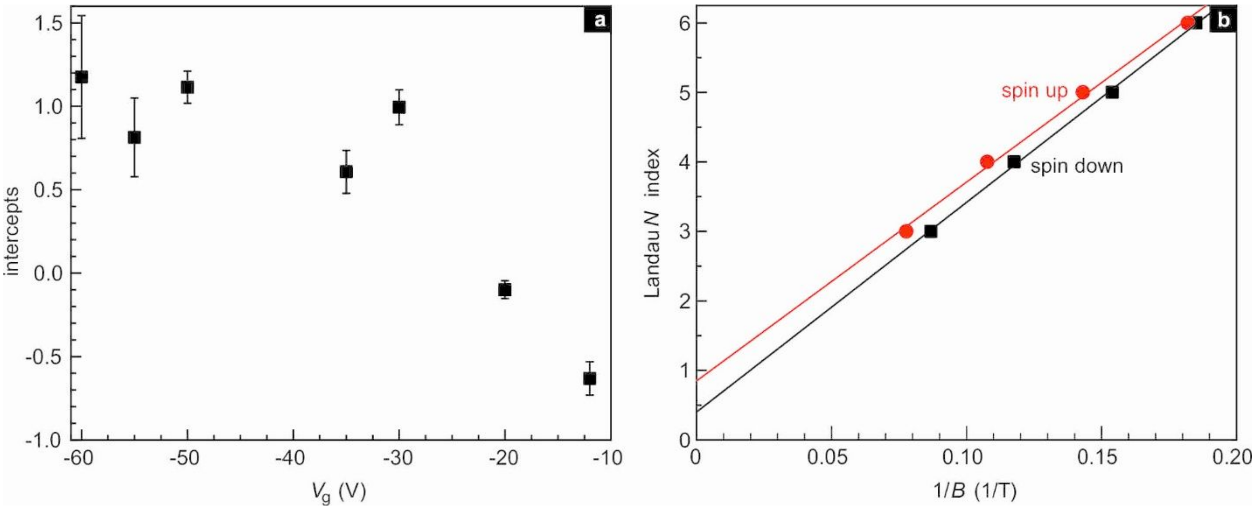

Fig. S10 | Determination of Berry phase and *g*-factor of holes a, Intercept of a linear fit to the oscillation extrema plotted in the inverse magnetic field versus Landau index plane as a function of the back-gate voltage. The data were recorded on device D1 (see Fig. 3d in the main text). **b**, Location of the oscillation minima corresponding to full occupation of the spin-up and spin-down branches of the Landau levels for V_g = -35 V. The data were obtained at 1.7 K.

11. The ν = 0 state and its evolution with magnetic field and temperature

Fig. S11 (recorded on device D2 with a $ZrTe_5$ layer thickness of about 7.6 nm) and S12 (recorded on device D1 with a $ZrTe_5$ layer thickness of about 6.5 nm) summarize longitudinal resistivity and Hall resistivity data as well as related quantities in order to illustrate how the ν = 0 incompressible ground state evolves with increasing magnetic field and temperature. Fig. S11a displays the lowest temperature data (1.7 K) for the maximum externally applied field of 13 T. Shubnikov-de Haas minima and plateaus in the Hall resistivity are identified by the corresponding filling factor. It is frequently beneficial to replot the data by transforming resistivities to conductivities in order to better bring out the ν = 0 state in the vicinity of the charge neutrality point. This has been done in panel b. The evolution of the Hall conductivity, σ_{xy}, and the longitudinal conductivity, σ_{xx}, with increasing magnetic field are illustrated in panels c and d. The width of $\nu = 0$ state barely changes with magnetic field, while the $\nu = 2$ state becomes wider as the magnetic field is raised.

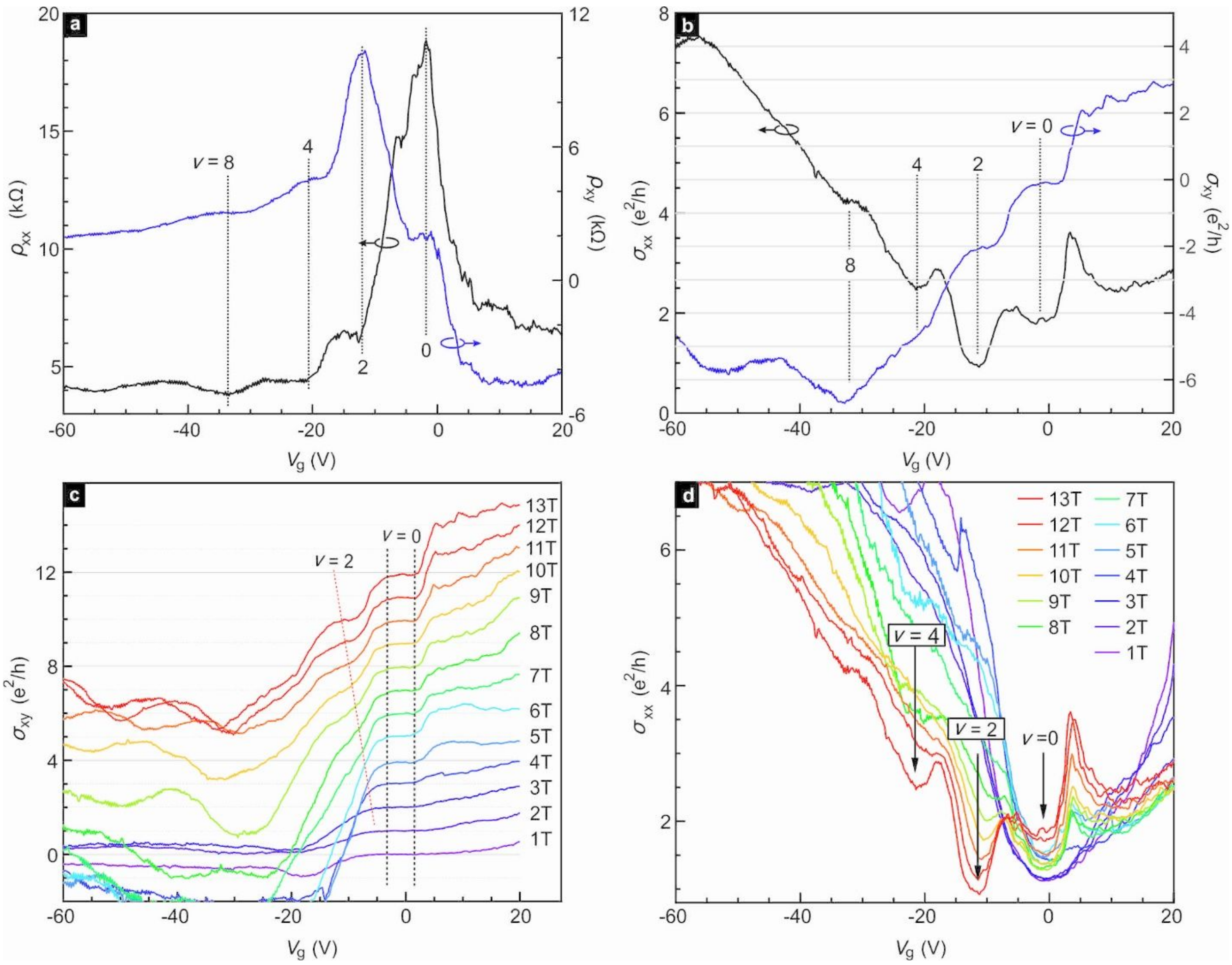

Fig. S11 | The ν = 0 state in device D2 with a 7.6 nm thick $ZrTe_5$ layer and its evolution with magnetic field. **a**, ρ_{xx} (black) and ρ_{xy} (blue) measured as a function of V_g at a fixed magnetic field of 13 T and T = 1.7 K. Minima and plateaus are marked by the filling factor they correspond to. **b**, σ_{xx} (black) and σ_{xy} (blue) obtained from the data in panel **a** using the inversion of the resistivity tensor. **c**, σ_{xy} as a function of gate voltage for different magnetic fields at T = 1.7 K. All curves are shifted vertically by one unit of e^2/h for clarity. Plateaus are marked with the corresponding filling factor. **d**, Same as in panel **c**, but for σ_{xx}.

Fig. S12 focuses on the temperature dependent behavior. The measurements plotted in this figure were taken on device D1. The longitudinal conductivity at 13 T as a function of back-gate voltage is plotted for temperatures between 1.7 K and 30 K. The Hall conductivity data across the same temperature range and at the same magnetic field (13 T) is shown in panel b,

whereas data recorded at a field of 1 T only is depicted in panel c. The plateau assigned to ν = 0 survives up to T > 100 K indicating the robustness of this incompressible state.

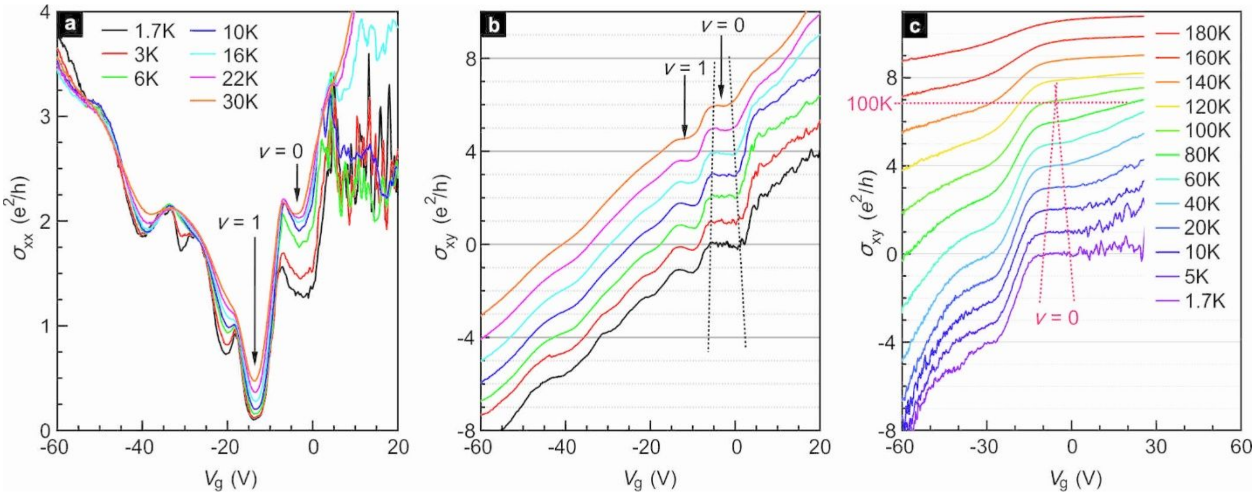

Fig. S12 | Temperature evolution of the ν = 0 incompressible ground state in device D1. **a**, σ_{xx} as a function of gate voltage for different temperatures at a fixed field B = 13 T. **b**, As in panel **a**, but for σ_{xy}. Adjacent curves are shifted vertically by one unit of e^2/h for clarity. The best developed plateaus are marked by the corresponding filling factor. **c**, Hall conductivity σ_{xy} as a function of gate voltage for temperatures ranging between 1.7 K and 180 K at a fixed, low magnetic field of 1 T.

12. Arrhenius analysis for the determination of the activation energy gap of the ν = 0 state

In order to extract the energy gap for thermal activation of charge carriers when the electronic system condenses in the incompressible ground state at filling ν = 0, the longitudinal resistance was measured as a function of temperature at fixed magnetic fields ranging from 0 T to 10 T. Such raw data sets obtained at small fields are shown in panel a of Fig. S13, whereas the data sets acquired at larger fields are shown in panel b. Above 2 T, curves nearly overlap with each other, indicating that the activation energy does not or is only weakly dependent on magnetic field. Activation energies are determined from an Arrhenius plot of the data with the inverse temperature as abscissae and ln(R_{xx}) as the ordinates.[8,9] An example of such an analysis is

shown for B = 2 T in panel c. A plot of the activation energies can be found in panel d of Fig. 4 in the main text.

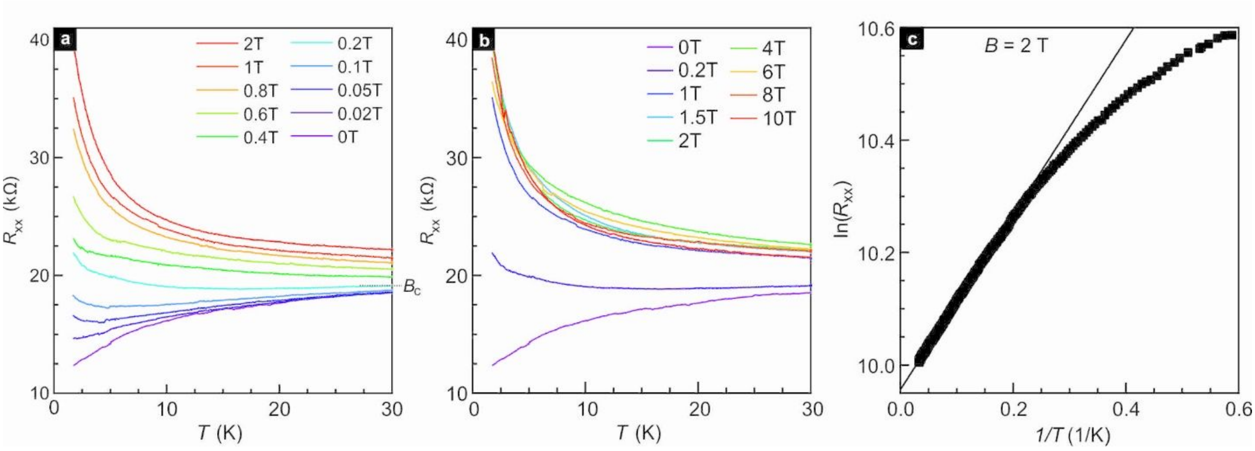

Fig. S13 | Temperature dependence of R_{xx} at ν = 0 and Arrhenius plot analysis of activation energy gap. a, R_{xx} as a function of temperature at different magnetic fields (ranging from 0 T to 2 T) at ν = 0. **b**, Same as in panel **a**, but for a larger field range R_{xx} (from 0 T to 10 T). **c**, Arrhenius plot showing ln (R_{xx}) as a function of T^{-1} for B = 2 T to estimate the energy activation gap at ν = 0. All data were taken on device D1.

13. Arrhenius analysis for the ν = 0, 1 and 2 ground states at 13 T

Here, as in the previous section, we attempt to determine the activation energy using an Arrhenius plot for minima that occur at filling 0, 1 and 2 when an external field of 13 T is applied. The results are summarized in Fig. S14. Fits yield the following activation energies for ν = 0, 1, and 2, respectively: 3.0 $\pm$ 0.2 K (~ 0.26 meV), 11.5 $\pm$ 2.3 K (~ 0.99 meV) and 3.2 $\pm$ 0.6 K (~ 0.28 meV).

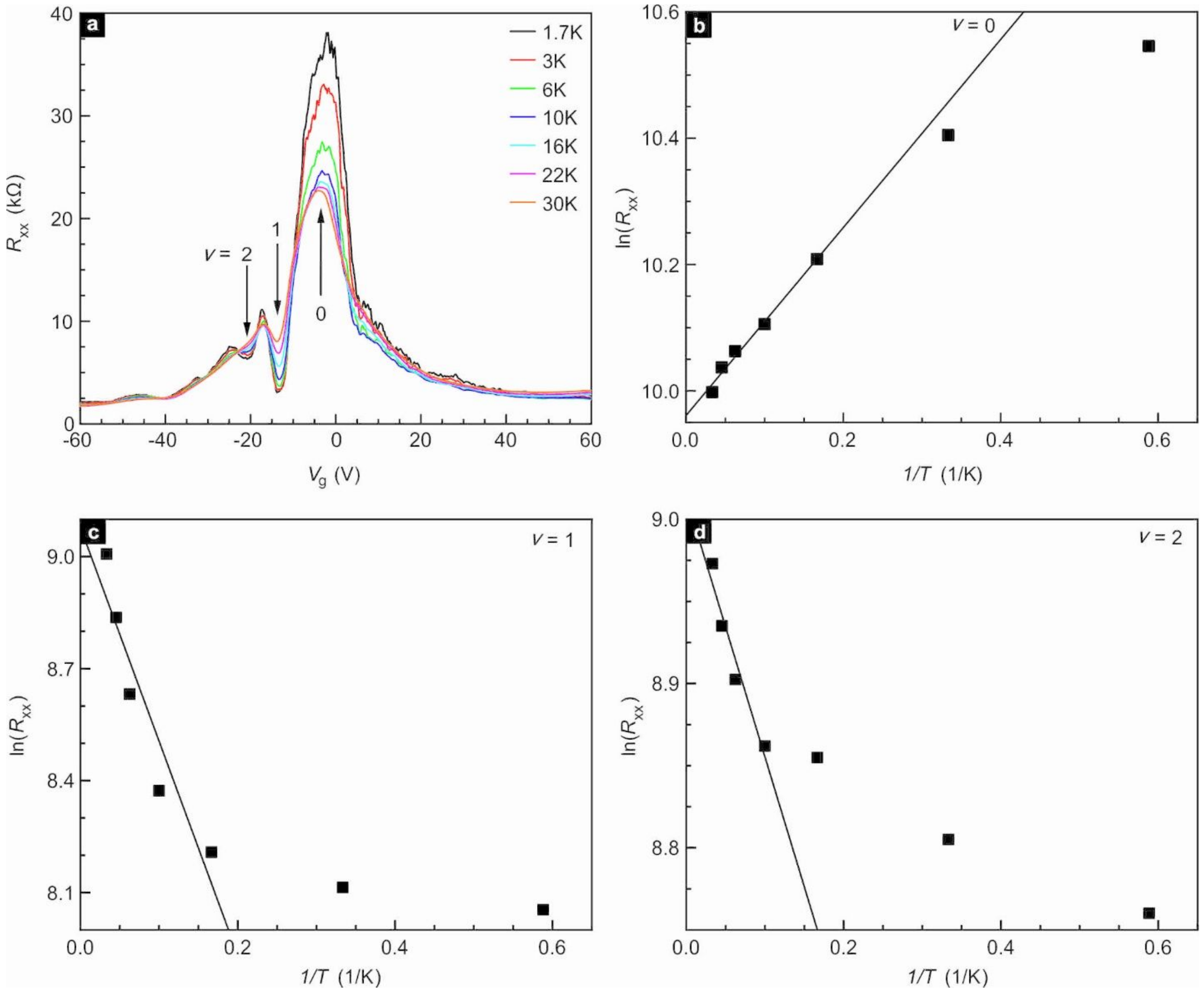

Fig. S14 | Arrhenius analysis of the activation energies at ν = 0, 1, 2 at B = 13 T. a, Temperature evolution of R_{xx} as a function of back-gate voltage. Minima at ν = 0, 1, and 2 are marked. **b, c and d**, Arrhenius plot of the temperature dependence of R_{xx} at filling ν = 0, 1, and 2, respectively. All data were taken on device D1.

14. Conductivity tensor correction considering in-plane anisotropy

In $ZrTe_5$, the large anisotropy of the Fermi surface is accompanied with a large difference of the effective mass and Fermi velocity among different crystal directions. This causes an anisotropy of the in-plane electrical transport properties[3,10,11]. In order to calculate the conductivity tensor elements $\sigma_{xx} = \rho_{xx}/(\rho_{xx}\rho_{yy} + \rho_{xy}^2)$ and $\sigma_{xy} = -\rho_{xy}/(\rho_{xx}\rho_{yy} + \rho_{xy}^2)$, the resistivity ρ_{yy} in the y direction (c axis) is needed. However, it is not possible to measure ρ_{yy} at the same time as ρ_{xx} in our device geometry. Therefore, we introduce the anisotropy ratio

$\alpha = \rho_{yy}/\rho_{xx}$ to estimate its impact on the conductivities. Previous measurements on bulk samples[3,5] reported anisotropy ratios larger than 10 using the expression $\frac{\rho_{yy}}{\rho_{xx}} \sim \frac{\mu_x}{\mu_y} \sim \frac{e\tau_x/m_x^*}{e\tau_y/m_y^*}$. In these samples, the chemical potential was very close to the Dirac point. Typical carrier densities were 10^{16} cm^{-3} and the mobilities were higher than 10^5cm^{2}V^{-1}s^{-1} indicating a low degree of impurity scattering. However, in an L-shaped $ZrTe_5$ device with a thickness of only 8 nm, comparable to the thicknesses of the samples discussed here, the anisotropy ratio was experimentally determined to be around 2 only.[10] Contrary to the previously mentioned bulk samples, the chemical potential in this L-shaped structure is pinned well inside the valence band as it exhibits a large carrier density (10^{18}cm^{-3}). The heavy hole doping and larger impurity scattering is likely caused by surface degradation in this device. Both may reduce the anisotropy ratio. The ratio applicable to our devices is most likely in between the value reported on bulk samples and the value extracted from the L-shaped device. In view of this uncertainty in α, we illustrate what happens to the conductivities for different anisotropy ratios (1, 2 and 4) in Fig. S15. For weakly developed quantum Hall effect features, the anisotropy correction causes a minor change in the value of each plateau in σ_{xy} as well as the minimum in σ_{xx}, because of the non-zero ρ_{xx}, but the overall behavior remains similar. For well-developed quantum Hall states with a clear plateau in ρ_{xy} and minima approaching zero in both ρ_{xx} and ρ_{yy}, the anisotropy correction would of course neither change the value nor position.

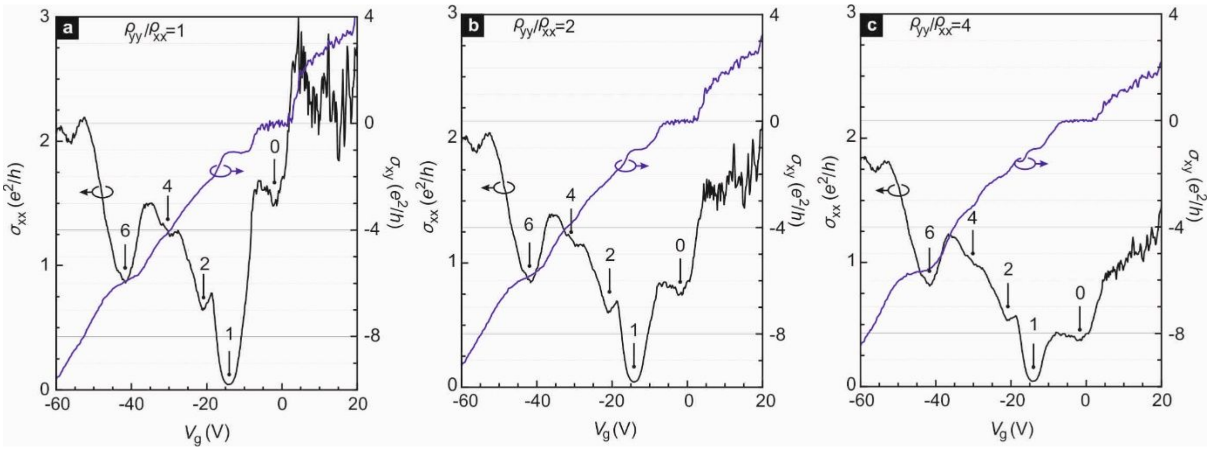

Fig. S15 | Conductivity tensor calculation with anisotropy ratio α = ρ_{yy}/ρ_{xx}=1, 2, and 4, respectively. a, σ_{xx} (black) and σ_{xy} (blue) obtained from Fig. 2a using the tensor inversion with anisotropy ratio α = 1. **b,** The same but for α = 2. **c**, Ditto but for α = 4.

15. Methods

Sample preparation. High-quality single crystals of $ZrTe_5$ were synthesized from high-purity elemental materials (99.9999% zirconium and 99.9999% tellurium) using either the tellurium flux method or chemical vapor transport[3,4]. The crystals are needle like and are placed on atomically flat hBN in order to achieve the best sample quality. The starting hBN material was purchased from HQ graphene and mechanically exfoliated using sticky tape to obtain thin h-BN flakes. They were put on top of a heavily doped Si substrate covered with a 300 nm thick dry thermal SiO_2. After spin-coating the substrate with a poly(methylmethacrylate) (PMMA), electron beam lithography was performed to make an electrode pattern on the hBN. The part of hBN exposed after development of the resist was etched away with a CHF_3/O_2 mixture in an Oxford ICP machine in order to produce buried contacts. Cr and Au metal were thermally evaporated with a total thickness that approximately matched the thickness of the hBN flake in order to minimize topography-induced strain when placing the $ZrTe_5$ crystal on top of the hBN. A typical thickness of the hBN flake was 20 nm. After lift-off, the sample was kept in a warm acetone bath (~60°C) for at least 4 hours to reduce polymer residues on the hBN. The sample was then transferred to a glove box filled with argon. The glove box atmosphere had a residual amount of H_2O and O_2 of less than 0.1 ppm. Few-layer $ZrTe_5$ was freshly cleaved onto a polydimethylsiloxane (PDMS) film inside the glovebox and transferred onto the hBN with pre-patterned contacts with the help of a homemade transfer stage. To avoid any exposure to ambient air, packaging and bonding of the device also proceeded inside the glove box with indium wires and a stereo microscope on a suitable chip carrier. The heavily doped Si substrate served as the back-gate. A schematic of the sample is illustrated in Fig. 1a and an optical image of a completed device is shown in panel b. With the help of a load lock arrangement, the sample was mounted into the sample rod and subsequently transferred to the cryostat without exposure to ambient air. The entire procedure avoided not only air exposure of the $ZrTe_5$ sample, but also any exposure to solvents or elevated temperatures, all of which would cause a degradation of the sample quality. Some devices were covered with an hBN on top, while others were not. We note that just capping the active device layer with an inert hBN layer before exposig the sample to ambient air did not prevent sample degradation. It seems that air is still able to leak into the heterointerface and large hole doping is induced. Therefore in this work we have implemented the much more cumbersome approach described above. We have investigated samples that in addition were covered with hBN and the results were not necessarily better. In some cases, the results were worse (in terms of the measured mobility) than without

encapsulation. This may be related to the significant number of bubbles that form at the interface between hBN and $ZrTe_5$. We stress that commonly used techniques to reduce bubbles such as heating cannot be applied here, since $ZrTe_5$ degrades even during mild temperature treatments.

Measurements. The thickness of the exfoliated $ZrTe_5$ crystal was measured using non-contact-mode atomic force microscopy (AFM) (NX-10 from Park System). The low-temperature magneto-transport measurements were performed in a dry cryostat system equipped with a superconducting magnet offering an axial field up to 14 T and a variable temperature insert that allows tuning the temperature between 1.7 K and 400 K. Low-frequency lock-in detection at 17.77 Hz was deployed for noise suppression to record the longitudinal and transverse resistance. Measurement currents ranged between 1 nA and 10 μA depending on the two-point resistance of the sample. A back-gate voltage was applied with the help of a Keithley 2400 Source Measure Unit.

16. References

1. Tang, F. *et al.* Multi-carrier transport in $ZrTe_5$ film. *Chinese Phys. B* **2018**, *27*, 087307.
2. Niu, J. *et al.* Electrical transport in nanothick $ZrTe_5$ sheets: From three to two dimensions. *Phys. Rev. B* **2017**, *95*, 035420.
3. Tang, F. *et al.* Three-dimensional quantum Hall effect and metal–insulator transition in $ZrTe_5$. *Nature* **2019**, *569*, 7757.
4. Shahi, P. *et al.* Bipolar Conduction as the Possible Origin of the Electronic Transition in Pentatellurides: Metallic vs Semiconducting Behavior. *Phys. Rev. X* **2018**, *8*, 021055.
5. Liu, Y. *et al.* Zeeman splitting and dynamical mass generation in Dirac semimetal $ZrTe_5$. *Nat. Commun.* **2016**, *7*, 12516.
6. Chen, R. Y. *et al.* Magnetoinfrared Spectroscopy of Landau Levels and Zeeman Splitting of Three-Dimensional Massless Dirac Fermions in $ZrTe_5$. *Phys. Rev. Lett.* **2015**, *115*, 176404.
7. Li, X.-B. *et al.* Experimental Observation of Topological Edge States at the Surface Step Edge of the Topological Insulator $ZrTe_5$. *Phys. Rev. Lett.* **2016**, *116*, 176803.
8. Zhao, Y., Cadden-Zimansky, P., Jiang, Z. & Kim, P. Symmetry breaking in the zero-energy landau level in bilayer graphene. *Phys. Rev. Lett.* **2010**, *104*, 066801.
9. Lin, B. C. *et al.* Observation of an Odd-Integer Quantum Hall Effect from Topological Surface States in Cd3As2. *Phys. Rev. Lett.* **2019**, *122*, 36602.
10. Qiu, G. *et al.* Observation of Optical and Electrical In-Plane Anisotropy in High-Mobility Few-Layer $ZrTe_5$. *Nano Lett.* **2016**, *16*, 7364–7369.
11. Chi, H. *et al.* Lifshitz transition mediated electronic transport anomaly in bulk $ZrTe_5$. *New J. Phys.* **2017**, *19*, 015005.
